# First Genome-Based Characterisation and Staphylococcal Enterotoxin Production Ability of Methicillin-Susceptible and Methicillin-Resistant *Staphylococcus aureus* Strains Isolated from Ready-to-Eat Foods in Algiers (Algeria)

**DOI:** 10.3390/toxins14110731

**Published:** 2022-10-25

**Authors:** Francesca Fanelli, Daniele Chieffi, Gyu-Sung Cho, Justyna Schubert, Omar Amine Mekhloufi, Jacek Bania, Charles M. A. P. Franz, Vincenzina Fusco

**Affiliations:** 1 National Research Council of Italy, Institute of Sciences of Food Production (CNR-ISPA), 70126 Bari, Italy; 2 Department of Microbiology and Biotechnology, Max Rubner-Institut, Hermann-Weigmann-Straße 1, 24103 Kiel, Germany; 3 Research and Development, Silantes GmbH, Gollierstrasse 70C, 80339 Munich, Germany; 4 Laboratory of Hygiene and Animal Pathology, Institute of Veterinary Sciences, University of Tiaret, Tiaret 14000, Algeria; 5 Department of Food Hygiene and Consumer Health Protection, Wrocław University of Environmental and Life Sciences, 50-375 Wrocław, Poland

**Keywords:** genomics, staphylococcal enterotoxins, *Staphylococcus aureus*, ready-to-eat food, virulence, antibiotic resistance, food safety, methicillin-resistant *Staphylococcus aureus* (MRSA), panton-valentine leukocidin (PVL), *luk*F-PV and *luk*S-PV genes

## Abstract

*Staphylococcus aureus* is a pathogenic microorganism of humans and animals, able to cause foodborne intoxication due to the production of staphylococcal enterotoxins (SEs) and to resist antibiotic treatment as in the case of methicillin-resistant *S. aureus* (MRSA). In this study, we performed a genomic characterisation of 12 genetically diverse *S. aureus* strains isolated from ready-to-eat foods in Algiers (Algeria). Moreover, their ability to produce some classical and new staphylococcal enterotoxins (SEs) was investigated. The 12 *S. aureus* strains resulted to belong to nine known sequence types (STs) and to the novel ST7199 and ST7200. Furthermore, *S. aureus* SA46 was assigned to the European clone MRSA-ST80-SCC*mec*-IV. The 12 strains showed a wide endowment of *se* and *sel* (staphylococcal enterotoxin-like toxin) genes (*sea*, *seb*, *sed*, *seg*, *seh*, *sei*, *selj*, *sek*, *sem*, *sen*, *seo*, *seq*, *ser*, *selu*2, *selw*, *selx*, *sey*, *sel*30; *ψent*1-*ψent*2), including variants and pseudogenes, and harboured the enterotoxin gene cluster (*egc*) types 1 and 5. Additionally, they produced various amounts of *SEA* (64.54–345.02 ng/mL), SEB (2871.28–14739.17 ng/mL), SED (322.70–398.94 ng/mL), SEH (not detectable–239.48 ng/mL), and SER (36,720.10–63,176.06 ng/mL) depending on their genotypes. The genetic determinants related to their phenotypic resistance to β-lactams (*bla*Z, *mec*A), ofloxacin (*gyr*A-S84L), erythromycin (*erm*B), lincomycin (*lmr*S), kanamycin (*aph*(3′)-III, *ant*(6)-I), and tetracyclin (*tet*(L), *tet*(38)) were also detected. A plethora of virulence-related genes, including major virulence genes such as the *tst* gene, determinant for the toxic shock syndrome toxin-1, and the *luk*F-PV and *luk*S-PV genes, encoding the panton-valentine leukocidin (PVL), were present in the *S. aureus* strains, highlighting their pathogenic potential. Furthermore, a phylogenomic reconstruction including worldwide foodborne *S. aureus* showed a clear clustering based on ST and geographical origin rather than the source of isolation.

## 1. Introduction

Despite the increased awareness of foodborne diseases and huge progress in food safety diagnostics, enterotoxigenic *Staphylococcus* (*S.*) *aureus* continues to cause staphylococcal food-poisoning (SFP) cases worldwide. Following the discovery of the classical enterotoxins (SEA-SEE), 33 staphylococcal enterotoxin (*se*) and staphylococcal enterotoxin-like toxin (*sel*) genes are known to date [1], highlighting the great enterotoxigenic potential of *S. aureus*. Moreover, this pathogenic bacterium may show resistance to several antibiotics [2] that may affect the antibiotic treatment when *S. aureus* infections occur. In particular, community-, livestock-, and healthcare-associated methicillin-resistant *S. aureus* (MRSA), even enterotoxigenic strains, are emerging in food products, animals used for foods, animal husbandry, and food production or processing workers [3].

Recently, we investigated the prevalence, enterotoxigenic potential, and antimicrobial resistance of 48 methicillin-susceptible *S. aureus* (MSSA) and MRSA isolated from 207 ready-to-eat foods randomly sampled from hotels, restaurants, fast foods, and pizzerias in Algiers, the capital of Algeria, during 2018 and 2019 [4]. The resulting *S. aureus* prevalence was 23.2% (48/207) [4]. For strain typing, intergenic spacer region (ISR) typing showed a higher discriminatory power than the staphylococcal enterotoxin gene (SEg) typing. Indeed, the same SEg type was found in *S. aureus* belonging to different ISR types. In particular, the 48 *S. aureus* strains were grouped in seven SEg types, while 12 ISR types were detected. Moreover, 2 out of the 48 *S. aureus* isolates, belonging to the same ISR type, were found to harbour the *mec*A gene that encodes for the penicillin-binding protein 2a (PBP2a), a transpeptidase protein with low affinity for most β-lactam antibiotics, including methicillin as well as oxacillin and cefoxitin [4,5]. Twelve strains, each belonging to one of the twelve detected ISR types, were subjected to antibiotic susceptibility testing. Some strains were resistant towards antimicrobials including benzylpenicillin, ofloxacin, erythromycin, lincomycin, tetracycline, kanamycin, oxacillin, and cefoxitin; the *mec*A-positive strain, being resistant to oxacillin and cefoxitin, was confirmed as MRSA, and two other tested strains showed multidrug resistance [4]. However, the performed molecular detection of *se* and *sel* genes did not permit to assess the presence of enterotoxin gene variants or pseudogenes, which may affect the enterotoxigenic activity and the virulence of the *S. aureus* strains, nor to distinguish among the *ψent*1/*ψent*2 pseudogenes and the *selu*2 gene in the enterotoxin gene cluster (*egc*) found in the *S. aureus* strains [4]. This drawback did not allow determining the *egc* type they harboured. This cluster plays a crucial role in the enterotoxigenicity of *S. aureus* since phylogenetic analyses indicated that potentially all the *se* and *sel* genes may derive from the *egc* [6], and, to date, at least eight *egc* types have been described in *S. aureus* [1,7]. Moreover, the presence of genetic determinants of antibiotic resistance, other than *mec*A, was not investigated in the 12 analysed strains [4]. Although some efforts are being made to characterise foodborne *S. aureus* in Africa, including the Algerian Country [8,9,10,11], specific data on *S. aureus* from ready-to-eat foods are scarce. Additionally, data on *S. aureus* molecular epidemiology are fairly increasing in the African continent, but studies are mainly performed on humans and animals rather than food isolates and MRSA rather than MSSA [12,13], underlying the need of collecting data on foodborne African isolates, including the Algerian Country. For all these reasons, the 12 Algerian MSSA and MRSA strains, representative of the different ISR types, were subjected to whole-genome sequencing. Herein, we reported a comprehensive genomic characterisation of these 12 genetically diverse *S. aureus*, exploring their genomic relatedness, investigating the genetic features in relation to their virulence and antibiotic resistance, and performing a phylogenomic analysis including worldwide foodborne *S. aureus* isolates. We also assessed the SEs production and detected all the *se* and *sel* genes known to date in order to improve the characterisation of foodborne *S. aureus* in the African continent.

## 2. Results

### 2.1. Genomic Statistics

The genomic features of *S. aureus* strains are reported in Supplementary Table S1. Genomes resulted in a median length of 2.7 Mbp and an average N50 value of 270 kbp. The average mol% GC content was 32.59%, with a median number of coding genes of 2606 and a coding density of 83.95%. The quality of the assemblies was excellent for all strains, with the exception of SA02, which resulted in only 95.5% completeness.

### 2.2. Multilocus Sequence and spa Typing

The sequence types (STs) of the 12 sequenced *S. aureus* strains are shown in Table 1, whereas the multilocus sequence type (MLST) allelic profiles are detailed in Supplementary Table S2. All strains belong to different STs, with the exception of strains SA02 and SA51, which both belong to ST5. Two novel STs have been deposited in PubMLST as ST7199 and ST7200: ST7199 differs from the known ST15 only for the novel *aro*E-1025 allele, whereas ST7200 differs from the known ST72 for the novel *yqi*L-954 allele.

Additionally, *spa* types of the 12 sequenced *S. aureus* strains are reported in Supplementary Table S3.

### 2.3. Phylogenetic Analysis

**Figure 1:**
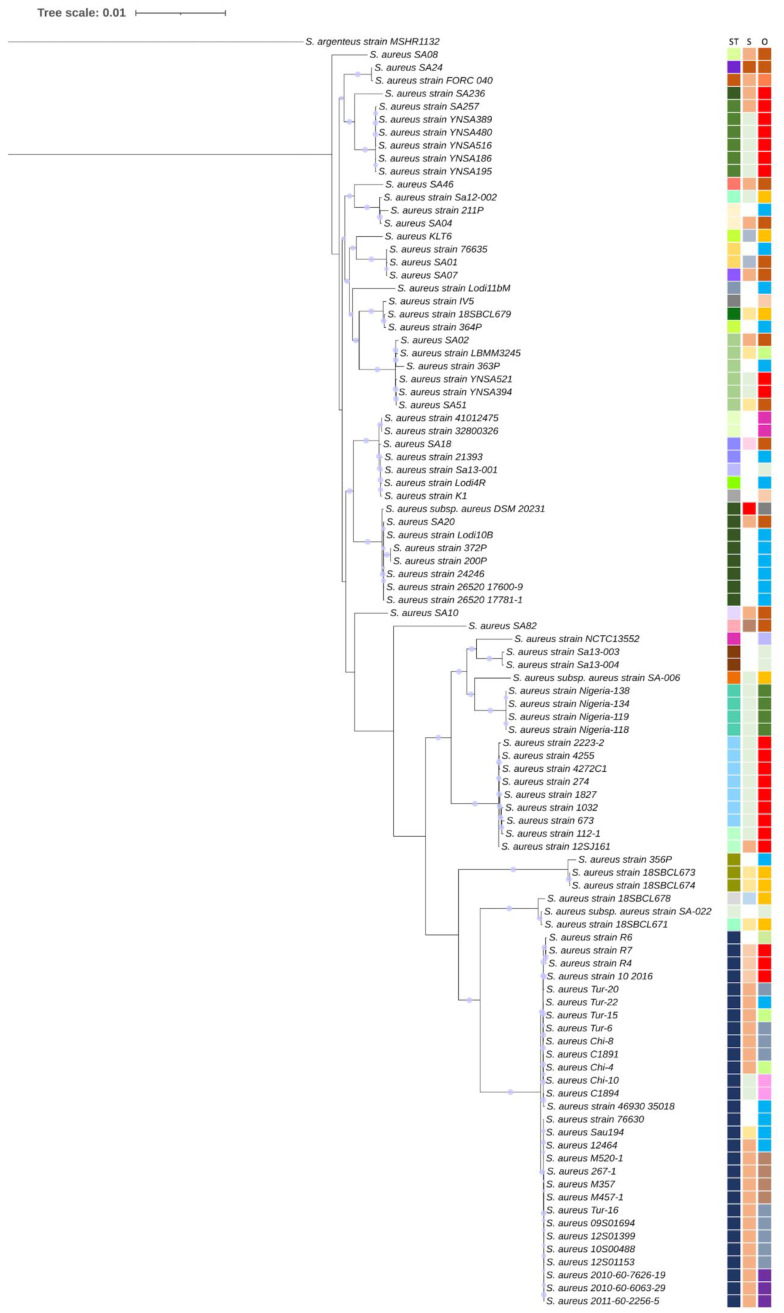
Phylogeny of *S. aureus* strains. Genome-based phylogenetic tree inferred by using maximum likelihood method RAxML with progressive refinement. *S. argenteus* MSHR1132 was used as outgroup. Support values are represented by scaled circles at each node. Tree is annotated with sequence type (ST), source of isolation (S), and geographical origin (O). See Supplementary Table S4 for detailed information.

A RAxML phylogenetic tree is reported in Figure 1. It also shows the ST, source of isolation (S), and geographical origin (O) of the analysed strains.

Globally, *S. aureus* strains are grouped in the tree mainly depending on the ST and geographical origin rather than the source of isolation, although a certain association appears for meat, milk, and dairy products. The grouping is clear for *S. aureus* isolates from China (red squares in the third column origin (O)), Nigeria (dark green squares in the third column O), and Italy (light blue in the third column O).

*S. aureus* strains sequenced in this study clustered on different branches of the tree. Those belonging to the same ST, namely SA02 and SA51, and those belonging to STs that differ by only a single allele, namely SA01 and SA07, closely grouped in the tree, whereas SA08, SA10, and SA82, belonging to STs that differ in numerous alleles, were individually located in the tree. The genetic divergence of SA82 from the other sequenced strains was also confirmed by ANI analysis, which reported average values of 98.3% between SA82 and the other sequenced strains, which, among them, share >99% of ANI (Supplementary Table S5).

### 2.4. Pan-Genome Analysis

The pan-genome of all the *S. aureus* isolates analysed in this study, which includes orthologues and unique genes, comprised a set of 7537 genes. The core genome (shared by >99% of *S. aureus* isolates), on the other hand, consisted of 1596 genes. The accessory genome (total core: genes in >2 isolates but not in all) consisted of 4059 genes, whereas the unique genome was composed of 1882 genes. The soft core, shell, and cloud genomes contained 261, 1323, and 4357 genes, respectively (Supplementary Table S6). The output of the Roary analysis is shown in Supplementary Figure S1, which confirms the clustering obtained by the phylogenetic analysis.

### 2.5. Enterotoxin Genes Presence and Localisation

The *se* and *sel* genes harboured by the 12 sequenced *S. aureus* strains, including variants and pseudogenes, are shown in Table 1. The nucleotide and amino acid sequences of the detected *se* and *sel* genes and predicted proteins, as well as the percentage of identity with previously reported reference sequences, are shown in Supplementary Table S7 [1,6,7,14,15,17,18,19,20,21,22,23,24,25,26,27,28,29,30,31]. 

*selw* is located on the chromosome. In SA02, SA51, SA18, and SA24, the *selw* gene was predicted as a pseudogene due to a frameshift mutation (SA02 and SA51) or an internal stop codon (SA18 and SA24). It should be pointed out that for SA82, SA08, and SA10 *selw*, the NCBI Prokaryotic Genome Annotation Pipeline (PGAP) annotated the predicted protein as *se* type 26 [1]. In this study, however, we retained the *selw* nomenclature as proposed since its discovery by Okumura et al. [32].

*selx* is also located on the chromosome in all the analysed strains.

In SA02, the enterotoxin gene cluster (*egc*) is located in a *S. aureus* pathogenicity island (SAPI) of νSaβ type I (SaPim3/n3), with *seg*, *sen*, *ψent*1-*ψent*2, *sei*, *sem*, and *seo* (J3P54_11775-11800) (*egc*1 [6]) (Supplementary Figure S2). This pathogenicity island harbours genes for the bi-component leukocidin LukED, serine protease genes *spl*ABCDEF with *spl*A being a pseudogene, genes for the restriction endonucleases M and S, and several transposase coding genes, some of which predicted as pseudogenes.

**Figure 2:**
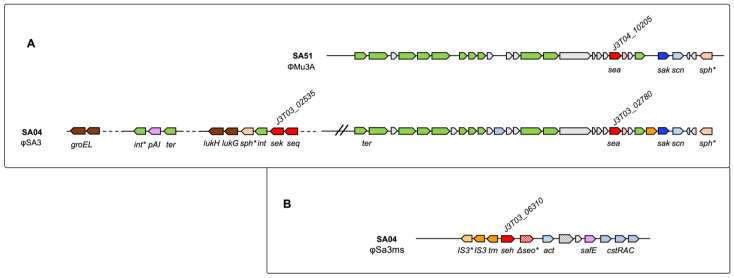
*sea* and *seh* prophagic regions in *S. aureus* SA04 and SA51 genomes. (**A**) ΦMu3A region comprising *sea* gene in SA51 genome and φSA3 region comprising *sea*, *sek*, and *seq* genes in SA04 genome. *se* genes are shown in red; *sak*: staphylokinase; *scn*: complement inhibitor SCIN-A; *sp*H: sphingomyelin phosphodiesterase; *int*: integrase; *p*AI: pathogenicity island family protein; *ter*: terminase; *luk*GH: bi-component leukocidin LukGH; *gro*EL: chaperonin GroEL; * indicates pseudogenes. (**B**) φSa3ms region comprising *seh* gene in SA04 genome. *act*: acetyltransferase; *saf*E: SafE family protein; *cst*R: persulfide-sensing transcriptional repressor CstR; *cst*A: persulfide response sulfurtransferase CstA; *cst*B: persulfide dioxygenase-sulfurtransferase CstB; * indicates pseudogenes.

SA08 harbours the *egc*5 (*seg*, *sen*, *selu*2, *sei*, *sem*, *seo*: KQP42_01815-01790) [7] in a νSaβ of type IV, with *luk*ED, serine protease genes, and a lantibiotic encoding gene cluster (KQP42_01860-01895) [33] (Supplementary Figure S2). SA24 harbours the *egc*5 (*seg*, *sen*, *selu*2, *sei*, *sem*, *seo*: J3135_04230-04205) located in a SAPI of νSaβ type I. We hypothesised a νSaβ of type III in SA82, but the contig is interrupted downstream the *egc*5 (*seg*, *sen*, *selu*2, *sei*, *sem*, *seo*: KQP39_04565-04540) (Supplementary Figure S2). In strain SA51, the *egc*1 (*seg*, *sen*, *ψent*1-*ψent*2, *sei*, *sem*, and *seo*: J3T04_07830-07855) is located in a νSaβ type I (SaPim3/n3) (Supplementary Figure S2). This genome also harbours *sea* (J3T04_10205) in a prophage region (ΦMu3A) (Figure 2A), harbouring the *sa*K gene and sphingomyelin phosphodiesterase pseudogene as a result of a frameshift mutation. In the genome of SA51, we also identified the *sel*30 gene (J3T04_08740). The plasmid location of this gene [1] appears to be confirmed by the nearby presence of *rep* pseudogene and the high homology (>99%) retrieved between a 17 kb flanking region comprising *sel*30 in *S. aureus* NCTC6135 and SA51 genome.

The *sea* gene is also present in the genome of SA04 in a prophage region of φSA3 type (Figure 2A). This large region includes 40 kb upstream *sea*, *seq*, and *sek* (Figure 2A), close to a site-specific integrase, a pseudogene for a sphingomyelin phosphodiesterase, a MBL fold metallo-hydrolase, the bi-component leukocidin LukGH, and the chaperonin GroEL.

In SA04, the genomic locus including the *seh* gene (J3T03_06310) is shown in Figure 2B and it is typical of a phage of φSa3ms type. Close to the *seh* gene, a pseudogene for the exotoxin *seo*, and the *cst* operon, which protects *S. aureus* from sulfide toxicity [34], can be identified.

In the SA18 genome, the *seb* gene is located in a pathogenicity island with 99.4% similarity with the SaPIivm60 described by Sato’o et al. [35].

**Figure 3:**
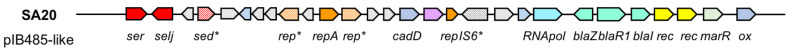
pIB485-like plasmid in *S. aureus* SA20 genome. Enterotoxin genes *ser* and *selj* are represented in red. *rep*: replication protein; *cad*D: cadmium resistance transporter CadD; RNA*pol*: RNA-directed DNA polymerase; *rec*: recombinase; IS6: IS6 family transposase; *bla*Z: beta-lactamase; *bla*R1: regulatory sensor/signal transducer BlaR1; *bla*I: beta-lactamase repressor BlaI; *mar*R: MarR family transcriptional regulator; *ox*: oxidoreductase; * indicates pseudogenes.

In the genome of strain SA20, the pseudogene *sed* (J3T02_03365) and the genes *ser* (J3T02_03360) and *selj* (J3T02_03355) are one by one located at the beginning of NODE_17, immediately followed by several *rep* protein genes, indicating the presence of a putative multiresistance pIB485-like plasmid [36] (Figure 3). This plasmid contains *bla*I, *bla*R1, and *bla*Z genes and shows a 100% nucleotide homology with the SAP048A plasmid (Accession: NC_019007.1), with an inversion due to the role of the two recombinase/invertase present in this locus (*bin* gene) [37,38].

In SA46, the *sey* gene (J3T00_13960) is located on the chromosome [7]. The genomic locus comprising *seh* (J3T00_044075), annotated as a pseudogene, also includes the staphyloferrin operon and three tandem-type lipoproteins.

Lastly, the sequences of allelic variants of *sea* [17], *sed* [18] and *selx* [7,15,16], different than those previously described, have been deposited in DDBJ/ENA/GenBank under the following accessions: seA-SA51 (ON205830.1); seD-SA20 (ON205834.1); selX-SA01 (ON205831.1); selX-SA08 (ON205832.1); selX-SA10 (ON205833.1); selX-SA46 (ON205835.1).

### 2.6. Antibiotic Resistance Genetic Determinants

Table 2 shows the antibiotic resistance profile, as assessed by Mekhloufi et al. [4], and the related genetic determinants identified in the *S. aureus* genomes analysed in the present study.

With the exception of SA02 and SA46, in all the other strains we identified *bla*Z, *bla*R1, and *bla*I genes, which encode for the penicillin-hydrolysing class A beta-lactamase BlaZ, the beta-lactam sensor/signal transducer BlaR1, and the penicillinase repressor BlaI, respectively. In *S. aureus*, the *bla*Z gene is carried by transposons [39], either located on a large plasmid or integrated into the bacterial chromosome. In strain SA24, we did not find any *rep* gene, which would suggest the presence of a plasmid. In contrast, such a *rep* gene was found in all the other *bla*Z-carrying strains. Moreover, the *bla*Z genomic context in SA24 does not appear to be similar to the other strains; indeed, it harbours few transposases, genes coding for YolD-like family proteins, integrases and recombinases, and the operon *pmt*ABCD, coding for the transporter responsible for the export of phenol-soluble modulins (PSMs), which modulate both biofilm formation and virulence in *S. aureus* [40]. In strain SA20, besides the presence of beta-lactam resistance genes, this region also harbours the pseudogene *sed*, as well as *selj* and *ser* genes, as described above (Figure 3).

In the *S. aureus* strain SA02 (resistant to ofloxacin, erythromycin, lincomycin, and tetracycline), we found an S84L substitution as a result of a mutation in the *gyr*A gene, which was reported to confer resistance to quinolones and fluoroquinolones [41]. The SA02 genome also harbours the *erm*B gene encoding a 23S rRNA (adenine(2058)-N(6))-dimethyltransferase associated with erythromycin resistance, a gene coding for the multidrug efflux MFS transporter LmrS involved in lincomycin resistance, as well as *tet*(L) and *tet*(38) genes, which are related to tetracycline resistance. *tet*(L) and *tet*(38) were closely located on a putative plasmid.

In the *S. aureus* strain SA18, we identified the *aph*(3′)-IIIa and *ant*(6)-I genes, which encode the aminoglycoside O-phosphotransferase APH(3′)-IIIa and the aminoglycoside nucleotidyltransferase ANT(6)-Ia, respectively, involved in the aminoglycoside resistance. These genes are located near *bla*Z, *bla*R1, and *bla*I on a mobile element. Furthermore, the genome of the strain SA18 also harbours the *tet*(L) and *tet*(38) genes involved in tetracycline resistance.

In addition, in the genome of the *S. aureus* strain SA46, which is resistant to benzylpenicillin, oxacillin, cefoxitin, and kanamycin, we identified the *aph*(3′)-IIIa and *ant*(6)-I genes. The *bla*Z gene was not present, whereas the genome harbours the *mec*A gene coding for the low-affinity penicillin binding protein (PBP) 2a responsible for the β-lactam resistance [42].

The strain SA51 was susceptible to the benzylpenicillin, as well as to the other tested antimicrobials (oxacillin, cefoxitin, gentamicin, kanamycin, tobramycin, ofloxacin, erythromycin, lincomycin, clindamycin, pristinamycin, linezolid, teicoplanin, vancomycin, tetracycline, fosfomycin, nitrofurantoin, fusidic acid, rifampicin, and co-trimoxazole [4]), even though the *bla*Z gene was shown to be present and intact in the genome of this strain.

### 2.7. Staphylococcal Chromosomal Cassette mec

In the genome of SA46, we identified a locus that has a homology of 88.74% with the staphylococcal chromosomal cassette *mec* (SCC*mec*) subtype-IVc(2B) and is identical to the clinical isolate GR2 (CP010402.1). The sequencing assembly has provided the dislocation of the SCC*mec* elements on two different contigs: *mec*A (J3T00_03665, NODE_6:211567..213573), *mec*R1 (J3T00_03670, NODE_6:213673..214659), IS1272 (J3T00_03680*, predicted as incomplete, NODE_6:214648..216490), *ccr*A2 (J3T00_04455, NODE_7:179619..180968), *ccr*B2 (NODE_7:180969..182618), and subtype-IVc(2B) (J3T00_04435, NODE_7:174448..175602). Although the prediction of SCC*mec*Finder classifies the SA46 *mec* complex as class B, the PAG pipeline predicted *mec*R1 as intact, with a sequence 100% identical to WP_001549960.1, whereas IS1272 was predicted as incomplete in the middle of the contig.

In SA04, genes with 92.2% of homology with *ccr*B1 (NODE_2:290655..292279) and 94.37% with *ccr*A1 (NODE_2:292301..293650) were predicted, but no whole SCC*mec* cassette was identified. In SA20, genes with 98.7%, 94.0%, and 99.8% of homology, respectively, were predicted with *ccr*A2 (NODE_20:3675..5024), *ccr*B2 (NODE_20:5046..6653), and subtype-IVa(2B): (NODE_10:93114..94604); moreover, in this case, no complete SCC*mec* cassette can be detected.

### 2.8. Virulence Determinants

**Figure 4:**
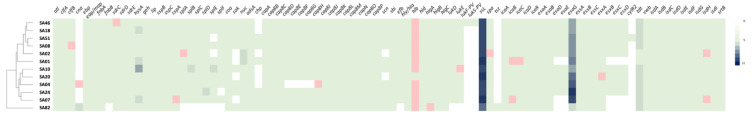
Virulence determinants in *S. aureus* genomes. Heatmap of virulence determinants in *S. aureus* genomes. Presence and numbers of genes are indicated by green colour intensity; absence of gene was indicated by a white square; pseudogenes are indicated as red-striped squares. Heatmap was visualised by using heatmapper web server [43] with average linkage as clustering method and Euclidean distance measurement method.

Comparative analysis of virulence determinants is described by the heatmap shown in Figure 4, whereas the list of genetic determinants is reported in Supplementary Table S8. All strains harbour a repertoire of genes coding for (a) proteins involved in the adherence, such as the autolysin gene, clumping factors *clf*A and *clf*B (this latter is a pseudogene in SA08), and elastin binding protein gene; (b) exoenzymes such as the hyaluronate lyase gene *hys*A, serine proteases, lipases, and the coagulase gene *vWbp* (von Willebrand factor-binding protein); (c) proteins involved in the immune modulation and in the heme uptake system; and (d) several exotoxins (Figure 4; Supplementary Table S8).

The staphylococcal superantigen-like (*ssl*) genes are arranged as tandem repeats in the genomic island α (*ssl*1–11) and in the immune evasion cluster 2 (IEC2, *ssl*12–14) on a vSaγ island on the bacterial chromosome [44]. This last island also comprises the *hyl* gene, which codes for the α-haemolysin, one fibrin-binding protein, and the *scn* gene coding for the staphylococcal complement inhibitor [45]. Only in *S. aureus* SA02 the contig is interrupted downstream *hyl*.

*S. aureus* SA82 is the only strain that harbours the *tst* gene coding for the toxic shock syndrome toxin (TSST-1), whereas the *luk*F-PV and *luk*S-PV genes coding for the Panton–Valentine leukocidin (PVL) are present on a bacteriophage φSa2 only in the genome of *S. aureus* SA46.

In the heatmap built on the presence or absence of genes or pseudogenes coding for virulence determinants, the strains are clustered into two groups, with SA82 in an individual clade, as already shown by the phylogenetic analysis (Figure 1). The SA82 genome, although harbouring the *tst* gene, lacked genes coding for (a) four out of five secreted virulence factors (EsxB, EsxC, EscD, and EsaD); (b) the soluble cytosolic protein EsaE of the type VII secretion system; and (c) the bi-component staphylococcal leukotoxins family LukDE [46]. It also missed (d) the *efb* gene coding for a fibrinogen binding protein; (e) the *fnb*B gene, encoding a fibronectin binding protein; and (f) all the genes coding for serine proteases. The *hlg*A gene, coding for the gamma haemolysin, is predicted to be a pseudogene. In contrast to all the other strains, the SA82 genome harbours two *cna* genes, encoding the collagen binding protein, which mediates bacterial adherence to collagen substrates and collagenous tissues, and is strongly associated with the pathogenesis of osteomyelitis and septic arthritis [47].

The *hlb* gene is interrupted due to a prophage integration in all strains with the exception of SA20.

### 2.9. Growth of S. aureus Strains in BHI + YE Broth and Production of Staphylococcal Enterotoxins

The concentrations of *S. aureus* strains SA04, SA18, SA20, SA46, and SA51 (harbouring *sea*, *seb*, *sed*, *seh*, and *ser* genes) at 0, 24, and 48 h of incubation in brain heart infusion broth supplemented with yeast extract (BHI + YE) are shown in Table 3. The pH values of the corresponding broth cultures are also reported (Table 3). At 0 h (immediately after the inoculum) the initial counts of all strains were similar (ranging between 6.38 ± 0.28 and 6.91 ± 0.29 Log cfu/mL). Major changes were observed after 24 h of incubation, when all bacterial numbers increased by more than 2 Log cfu/mL (ranging between 8.97 ± 0.09 and 9.34 ± 0.23 Log cfu/mL). Concentrations after 48 h of incubation were found at similar levels to those found after 24 h of incubation (ranging between 9.08 ± 0.13 and 9.44 ± 0.28).

With the exception of SA46, for which the SEH production was 1.76 ± 2.26 ng/mL at 24 h and not detectable at 48 h, all the other strains produced higher amounts of SEA, SEB, SED, SEH and SER at both 24 h and 48 h of incubation in the BHI + YE broth, as shown in Table 4.

### 2.10. Expression of the seh Gene

**Figure 5:**
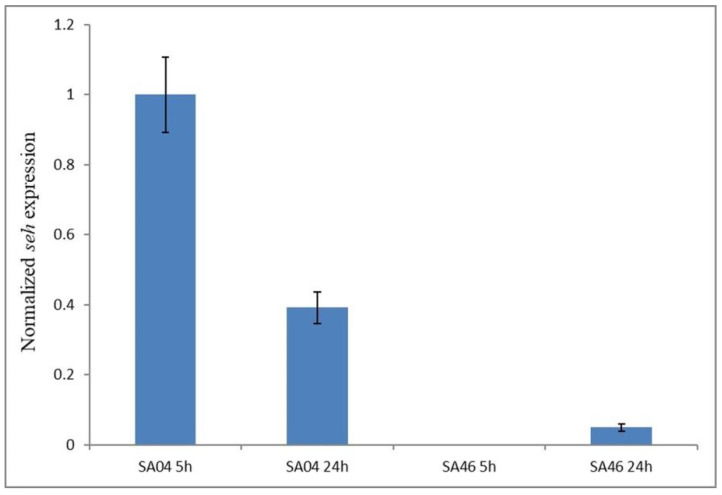
*seh* relative gene expression. *seh* relative gene expression in *S. aureus* SA04 and SA46 at 5 h and 24 h of incubation in BHI + YE broth.

The relative expression of the *seh* gene was evaluated in SA46 as well as in the other *seh*-positive strain SA04, at 5 and 24 h of incubation in the BHI + YE broth. As shown in Figure 5, in SA46 no transcription of the *seh* gene was observed at 5 h of incubation, whereas a very low transcription level compared to SA04 (0.13 fold) was detected at 24 h of incubation. In SA04 *seh* expression decreased at 24 h compared to that at 5 h (0.39-fold).

## 3. Discussion

In this study, we performed a comprehensive genomic analysis of 12 *S. aureus* strains isolated from 207 Algerian ready-to-eat foods to explore their genetic relatedness, gain further data on *S. aureus* molecular epidemiology, and investigate their virulence and antibiotic resistance genetic determinants that may help to better understand the features of foodborne *S. aureus* in Algeria as well as in the African continent.

Two novel alleles, *aro*E-1025 and *yqi*L-954, and two novel STs, 7199 and 7200, were identified and deposited in the PubMLST public database, thus contributing as new suitable resources for monitoring the global and local epidemiology of *S. aureus*. Among the other nine detected STs, six have been previously reported in Algeria, i.e., ST1-, ST8-, ST15-, ST22-, and ST97-MSSA clones as well as the ST80-MRSA clone [48,49], whereas ST5-, ST25-, and ST101-MSSA clones have been detected in other African countries [12,50,51]. In our study, the detection of a strain that is a member of the ST80-MRSA-SCC*mec*-IV lineage, also referred to as the European clone [52], is of particular interest since it has been described as having an alarming dissemination in Algeria [49]. It is worth mentioning that the clones herein found in Algerian ready-to-eat foods have been also found in humans (ST1-, ST5-, ST22-, ST25-, ST97-, and ST101-MSSA and ST80-MRSA-SCC*mec*-IV [12,49,50,51]) and animals (ST1-, ST8-, ST15-, ST22-, and ST97-MSSA and ST80-MRSA-SCC*mec*-IV [48,49]). This confirms the complexity of *S. aureus* epidemiology and highlights the fact that ready-to-eat foods, besides posing a risk to consumers for the onset of SFP, may also serve as means for *S. aureus* dissemination via food ingestion or handling, as pointed out also by other authors [3,53,54].

Considering the role of *S. aureus* in foodborne diseases due to the production of SEs [3], understanding the enterotoxigenic capabilities of circulating strains is a pivotal issue. Following the discovery of the classical SEs (SEA-SEE) [55,56], the number of the newly discovered SEs and SEls is constantly increasing. In particular, a total of 33 *se* and *sel* genes (*sea*-*see*; *seg*-*selu*; *selu*2; *selv*-*selz*; *sel*27-*sel*33) are known to date, including the last five *sels* recently reported (*sel*29 pseudogene; *sel*30-*sel*33) [1], thus indicating the wide enterotoxigenic potential of *S. aureus*. To the best of our knowledge, this is the first study searching for all the *se* and *sel* genes known to date and reporting the *sel*30 gene in Algerian *S. aureus* isolates from food after its recent discovery [1]. The gene, detected in SA51, shares 100% homology with the sequence reported by Dicks et al. [1]. Recently, some authors made efforts to gain data on the enterotoxigenic potential of foodborne *S. aureus* in Algeria, searching for some of the known *se* and *sel* genes (*sea*-*see*; *seg*-*sek*; *selp* and *ser*) [8,10,11], as well as the presence of the enterotoxin gene cluster (*egc*) [8]. These authors found the *sea*, *seb*, *sec*, *see*, *seg, seh*, *sei*, *selj*, *sek*, *selp*, *ser*, and the *egc*, although, in the latter case, no information on the actual *egc* gene content and the *egc*-type was provided [8,10,11]. We should remark that for the assessment of *S. aureus* enterotoxigenicity, not only the presence of *se* and *sel* genes but also the evaluation of genetic variants and the presence of pseudogenes, as herein performed, should be considered, since they may directly affect the enterotoxin production and virulence of the hosting *S. aureus* strains.

According to the results of our genome analysis, *selw* and *selx* genes were identified in all the sequenced strains, both located in the chromosome, in line with other studies [15,32]. *selw* was predicted as a pseudogene in 4 of 12 *S. aureus* strains, confirming that this gene, likely due to its chromosomal localisation, has a high rate of pseudogenisation [1]. Otherwise, no pseudogenes for *selx* were herein detected, corroborating previous reports in which an overall low prevalence of *selx* pseudogenes was observed, probably linked only to specific *S. aureus* lineages [1,16].

Most of the SFP diseases are associated with the classical SEs (SEA-SEE) [57]; furthermore, SEH has been found as a causative agent of SFP outbreaks [25,58]. Moreover, other newly discovered SEs such as SER and those belonging to the enterotoxin gene cluster (*egc*) are being reported with increasing evidence to have a possible role in foodborne intoxications [59,60,61].

The *sea* gene belonging to allele class 1 (*sea*1) [17] was identified in SA04, whereas SA51 harbours the *sea*2, a single-nucleotide sequence variant of the *sea*2 reported by Borst and Betley [17], that leads to no amino acid change in the predicted protein sequence (Table 1; Supplementary Table S7). SA04 produced higher amounts of SEA (156.04 ± 36.58–345.02 ± 62.67 ng/mL) compared with those produced by SA51 (64.54 ± 9.29–74.91 ± 7.36 ng/mL), being 2.4- and 4.6-folds higher at 24 h and 48 h, respectively. SEA, responsible for the majority of the SFP cases (about 80%) [57], is reported to cause human intoxication symptoms at a low dose of about 20–200 ng [62,63], and it is frequently detected in small amounts (ranging from 0.015 ng to >6 ng per gram or millilitre of food) in foods involved in SFP outbreaks [62,63,64,65]. These findings highlight the potential pathogenicity that our SEA-producing *S. aureus* strains may have in the contest of SFP. As regard the difference in SEA production observed in SA04 and SA51, it has been associated, by Borst and Betley [17], to the region upstream the translational start sites of *sea*. This region in SA04 is identical to that of the high-SEA-producing *S. aureus* FRI100 (harbouring *sea* allele class 1), whereas that in SA51 is identical to that of the low-SEA-producing *S. aureus* FRI281A (harbouring *sea* allele class 2) (Supplementary Table S7). However, the dissimilarities in the SEA amounts registered for SA04 and SA51 were not so pronounced as for *S. aureus* FRI100 and *S. aureus* FRI281A, since also other loci may affect the phage-mediated mechanism responsible for SEA expression [17].

*seb*v1 was identified in *S. aureus* SA18. This strain was able to produce SEB (2871.28 ± 811.09–14739.17 ± 5077.70 ng/mL) corroborating the findings of Johler et al. [18] who reported that all the described *seb* variants (*seb*v1-v4) are expressed, leading to the SEB production. In the literature, SEB-related SFPs are poorly documented; nevertheless, in a described outbreak involving boiled eggs, a high SEB concentration, greater than 1000 ng/g [66] and thus similar to that produced in the BHI + YE broth by our *S. aureus* strain, was reported. Unlike *sea*, differences in the *seb* upstream region were reported as not directly correlated with the SEB production level, which, on the other hand, may be linked to the type of the *seb*-harbouring SAPIs. Interestingly our *seb*-positive strain harbours a SAPI that has 99.4% similarity with SaPIivm60, which has been associated with strong SEB production by Sato’o et al. [35].

In SA20, *sed* was predicted as a pseudogene, which differs for a nucleotide substitution (leading to 1 amino-acid substitution) from the truncated *sed* described by Johler et al. [18] (Table 1; Supplementary Table S7). SA20 was able to produce SED (322.70 ± 41.17–398.94 ± 64.79 ng/mL), predicted as shorter than the intact protein (179 compared with the 258 amino acids of the intact SED) [18]. We should highlight that the prediction of the toxic properties based on the changes in the length and/or amino acid sequence of the SEs may be difficult, considering that the mechanism of action of SEs is not fully elucidated [18]. However, it should be highlighted that the truncated SED may have an impaired toxic function, resulting in a reduced virulence of the producing *S. aureus* strain. Low (0.052 ng/g [67]) to high (>200 ng/g [63]) concentrations of SED have been detected in foods involved in SFP outbreaks, although, to the best of our knowledge, no estimation of the oral dose capable of causing human symptoms has been reported. Some *S. aureus* strains harbouring the truncated *sed* gene were found either to not produce the relevant protein at detectable levels or even to not transcribe this pseudogene [18,68]. Nevertheless, Johler et al. [18] reported that certain *S. aureus* harbouring the truncated *sed* were able to produce the SED protein, being in line with our finding, although they found that the amounts were far lower than those produced by strains harbouring the intact *sed* gene.

*selj* and *ser* genes were also present in SA20 and located along with *sed* on the same putative pIB485-like plasmid as previously described [36]. Although also *ser* pseudogenes have been recently reported [7,69], SA20 harbours an intact *ser* gene and was able to produce SER (36,720.10 ± 6272.32–63,176.06 ± 14,422.93 ng/mL) in amounts more than 100-folds higher than SED. Interestingly, *S. aureus* strains harbouring the plasmid-related *sed*, *selj*, and *ser* genes have been isolated from seven SFP outbreaks in Japan [60], and their ability to produce SER in BHI + YE (523–2925 ng/mL) has been demonstrated, suggesting that SER may have played a possible role in the onset of the described SFPs [60].

The *seh* gene was detected as intact or as a pseudogene in two of our sequenced strains. SA04, harbouring the intact *seh*, was able to produce the enterotoxin SEH (221.76 ± 38.15–239.48 ± 84.83 ng/mL) (along with SEA, as discussed above), and, notably, the nucleotide sequence was identical to that of the *seh* gene found in *S. aureus* strains that caused a SEH-mediated SFP outbreak ([25]; Supplementary Table S7), indicating, therefore, the potential virulence of this strain. In particular, in the reported outbreak, a concentration of SEH equal to 55.5 ng/g was estimated in the consumed mashed potato made with raw milk, and the tested *S. aureus* strains, isolated from these leftovers and the farm bulk raw milk, were able to produce SEH in concentrations similar (96–108 ng/mL) to that produced by our SA04 strain, when grown in a microbial broth [25]. The *seh* pseudogene was instead detected in the SA46 strain belonging to the European MRSA-ST80-SCC*mec*-IV clone, corroborating the study of Dicks et al. [1] who recently reported this pseudogene as strongly associated with the MRSA-ST80-SCC*mec*-IV clone. SA46 also harboured the *sey* gene, whose sequence was previously detected in a ST80 strain [24]. SA46 produced SEH at almost undetectable levels. To investigate if the difference in the SEH amounts produced by SA04 and SA46 may depend on differences in *seh* expression, we assessed it taking into account that, in experimental conditions similar to those herein applied, the *seh* gene expression occurs during the exponential phase of growth [70]. The expression analysis detected no transcripts at 5 h for SA46, whereas at 24 h, their levels were much lower than those of the intact *seh* gene, harboured by SA04, confirming that, in this case, the failure of the SEH production in SA46 depends on transcriptional regulation. This is interesting since certain *se* pseudogenes may be transcribed at levels similar to the intact *se* genes, as found by Lis et al. [68], suggesting that the protein synthesis failure may occur later, at the translational level.

Although the involvement of MRSA in SFP has been reported [71] based on these findings and considering the relative little assortment of *se* and *sel* genes (*seh* pseudogene, *sey*, *selx*, and *selw*), it seems that our MRSA strain (SA46) may play a minor role in the onset of SFP compared with the other enterotoxin producing MSSA strains herein detected. A previous study found one *S. aureus* strain belonging to the European clone carrying the *egc*-related genes *seg*, *sei*, *sem*, *sen*, *seo*, and *selu* [72], whereas *seb* and *sek* were harboured by atypical MRSA-ST80-SCC*mec*-IV *pvl*-negative isolates [73]. Therefore, our findings may help to enrich the knowledge regarding the endowment of *se* and *sel* genes within the European clone in order to improve the understanding of the enterotoxigenic potential of this lineage.

The *egc* was found in 5 out of the 12 sequenced *S. aureus* strains, corroborating previous investigations that reported that *egc*-related genes are among the most prevalent genes in foodborne *S. aureus* isolates [74]. Beyond its correlation to SFP, the *egc* presence seems to be relevant also in other clinical conditions, such as chronic infections in cystic fibrosis patients [75]. This cluster may harbour several *se* and *sel* genes (i.e., *seo*, *sem*, *sei*, *selu*, *sen*, *seg*, *selu*2, *selv*, and *sel*33) and pseudogenes (*ψent*1-*ψent*2) in different combinations, and, to date, at least eight *egc* types have been described in *S. aureus* [1,7]. Two of our strains harbour the *egc*1 (*seg*, *sei*, *sem*, *sen*, *seo*, and *ψent*1-*ψent*2) and three strains harbour the *egc*5 (*seg*, *sei*, *sem*, *sen*, *seo*, and *selu*2). Both these types have been detected in foodborne *S. aureus* isolates [7], but there is still scarce knowledge on their actual prevalence mostly due to the fact that *egc*5 was only recently described [7].

The emergence of antimicrobial resistance in *S. aureus* is a clinical and public health challenge due to the reduction of the antimicrobial assortment that is effective when *S. aureus* infections occur. In addition to MRSA, whose presence has been globally reported in various ecological niches including African food, animals, and humans [13,76], also multidrug-resistant MSSA, typically showing resistance to three or more antimicrobials of different classes, as herein reported for SA02 and SA18, are being detected in various sources comprising food products (especially animal-derived unprocessed products) in Africa [23,77,78,79] and worldwide [54,80,81,82]. Genome analysis allowed us to detect the antimicrobial resistance genes related to the phenotypic resistance profile shown by our sequenced *S. aureus* strains. In particular, the *bla*Z gene and regulatory system composed of *bla*R1 and *bla*I that we found in 10 out of the 12 sequenced strains represent the genetic mechanism conferring resistance to benzylpenicillin and other penicillinase-labile penicillins. We should emphasise that in *S. aureus* SA51 we found the *bla* locus (*bla*Z, *bla*R1, and *bla*I), although it was reported as benzylpenicillin-susceptible. Since 2012, CLSI acknowledged that penicillin-susceptible results should be further investigated before being confirmed, performing additional phenotypic tests to detect β-lactamase production (i.e., nitrocefin-based tests and/or penicillin zone edge) [83]. However, these phenotypic methods were described as less sensitive compared with the molecular detection of the *bla*Z gene, and, therefore, different authors considered the latter the gold standard method for β-lactamase detection in *S. aureus* [84,85].

*bla*Z is also frequently found in MRSA [86,87], including the ST80-MRSA-SCC*mec*-IV lineage as previously reported for most of the analysed isolates by Monecke et al. [86]. Our ST80-MRSA-SCC*mec*-IV strain, SA46, although harbouring the *mec*A gene conferring resistance to β-lactam antimicrobials [87] such as benzylpenicillin, oxacillin, and cefoxitin, does not harbour the *bla*Z gene, and, in Africa (in Cape Verde), a similar feature was recently reported for other MRSA isolates belonging to the ST5-SCC*mec*-VI lineage [88]. Although the *bla* system is involved in the regulation of the *mec*A expression [89], it has been elucidated that the loss of the *bla* system may allow the constitutive expression of the *mec*A gene, conferring β-lactam resistance in SCC*mec*-IV harbouring MRSA [89].

It has also been reported that 91.6% of ST80-MRSA isolates are resistant to kanamycin, and the kanamycin-resistance gene *aph*(3′)-III has been detected in the ST80-MRSA clone [90], which is consistent with our finding. Beyond *aph*(3′)-IIIa, also the other detected genes conferring resistance to aminoglycosides (*ant*(6)-I), lincosamides (*lmr*S), tetracyclines (*tet*(L), *tet*(38)), macrolides (*erm*B), quinolones, and fluroquinolones (*gyr*A-S84L) have been previously reported in *S. aureus* isolates [91,92,93,94]. Nevertheless, a paucity of studies detected a range of antimicrobial resistance determinants in foodborne *S. aureus* in Africa, and, in particular, only few of the aforementioned genes were previously reported in Tunisia (*aph*(3′)-IIIa, *tet*(L) [95]), Kenya (*aph*(3′)-IIIa [96]), and South Africa (*aph* (3′)-1-IIIa, *gyr*A-S84L [77,97]). Therefore, the present study helps to further expand the knowledge on the genetic determinants harboured by antimicrobial-resistant *S. aureus* isolated from food in the African continent.

The results of the genomic analysis highlighted a plethora of different virulence determinants in all the sequenced strains, involved in adhesion, colonisation, spreading, and immune modulation. Adherence is the first process involved in the pathogenicity of *S. aureus*. It is determined by the expression of several genes such as that coding for the autolysin, which mediates adherence to immobilised fibrinogen and fibronectin [98], the clumping factors *clf*A and *clf*B that bind to different sites in fibrinogen, and fibronectin binding proteins A and B, which facilitate the attachment of *S. aureus* to host cells [99].These genes are present in all the sequenced strains. Exoenzymes such as the hyaluronate lyase coded by *hys*A are required for the degradation of hyaluronic acid, which contributes to the local dissolution of the extracellular matrix, whereas vWbp enables the bacteria to disseminate and resist to opsonophagocytic clearance by host immune cells [100]. Among the exotoxins, haemolysin genes were retrieved in all the strains. The *hlb* gene, which codes for a sphingomyelinase that cleaves sphingomyelin into phosphocholine and ceramide [101], was a pseudogene in all strains, with the exception of SA20. The interruption of this gene is caused by the integration of a prophage of Sa3int type. Loss and reintegration of this phage are correlated with the transmission of *S. aureus* from humans to livestock and vice versa [102]. The restoration of the *hlb* gene, which occurred in SA20, might be relevant for important specific infections [103,104].

Panton-Valentine leukocidin (PVL) is a major virulence factor of *S. aureus*, although it presents in less than 5% of *S. aureus* strains. It is a two-component toxin that induces pore formation in the leukocyte cell membrane complement receptors [105]. The results of our genomic analysis detected *pvl* genes only in *S. aureus* SA46, harbouring SCC*mec* of type IV. PVL has been proposed as a marker of CA-MRSA [106], although PVL-negative CA-MRSA clones have been reported [52].

According to the international working group on the Classification of Staphylococcal Cassette Chromosome [107], the genomic sequence of SA46 meets the criteria for defining an SCC*mec*: (i) carriage of *mec*A in a *mec* gene complex; (ii) presence of *ccr* gene(s) (*ccrAB* and/or *ccrC*) in the *ccr* gene complex; (iii) integration at a specific site in the staphylococcal chromosome, designated as the integration site sequence (ISS) for SCC, which serves as a target for *ccr-*mediated recombination; and (iv) presence of flanking direct repeat sequences containing the ISS. SCC*mec* of SA46 was predicted as SCC*mec* subtype-IVc(2B), although the B class of the *mec* complex is characterised by the presence of *mec*A and a truncated *mec*R1 resulting from the insertion of IS1272 upstream *mec*A [108]. On the contrary, the annotation of this genome returned an intact *mec*R1 and an incomplete IS1272, in the middle of a contig. Indeed, the whole cassette was dislocated in two separate nodes. This failure in reconstructing the entire SCC*mec* as the result of short-read sequencing technology is due to the multiple insertion sequences, which cause difficulties in the assembly.

To date, only two studies performed a genomic analysis of Algerian *S. aureus* strains: one by Mairi et al. [109], who reported the first occurrence of the “Maltese clone” (CC5-MRSA-IV-SCC*fus*) in Bat Guano, and a second by Aouati et al. [110], who uncovered the emergence of MRSA SCC*mec*-III Mercury in clinical isolates in Eastern Algeria. Other sequence read archives (SRAs) obtained by sequencing *S. aureus* isolated from Algerian dairy products were deposited in NCBI (Accession ERS4338951-ERS4338960).

This is the first genomic study performed on Algerian foodborne *S. aureus* strains and, to the best of our knowledge, the first comparative and phylogenetic reconstruction of foodborne *S. aureus* of worldwide origin. According to our data, there is a clear phylogenetic association of *S. aureus*, based on the ST or the geographical origin, rather than the source of isolation. This reflects the dynamics and evolution of the *S. aureus* population worldwide. The arising grouping based on the food source might be an effect of the sample collection rather than a clustering based on the use of a specific ecological niche.

The Algerian isolates are dispersed in the phylogenetic tree with the exception of SA51 and SA02, which both belong to the ST5, and SA01 and SA07, which differ for the *aro*E allele in the MLST. From an evolutionary point of view, SA08 is the less recent strain, whereas SA82, which is individually located in all the cluster analyses we have performed, is the most recent. However, such phylogenetic reconstructions are affected by the fact that most strains, although sharing the same geographical origin, have been isolated from different food products, some generically indicated as food, and no information is available on the manufacturing process nor on the origin of raw material used, thus hindering an exhaustive reconstruction of phylogenetic and epidemiological maps.

This underlines the urgent need for genomic studies on foodborne *S. aureus* in Algeria to have more detailed insights regarding the structure of the *S. aureus* population as well as the harboured genetic determinants of virulence and antibiotic resistance, in order to be able to estimate the public health burden related to the spread of this pathogen in food.

## 4. Conclusions

A comprehensive genomic characterisation and the production of some classical and newly described staphylococcal enterotoxins of 12 Algerian *S. aureus* strains, previously isolated from 207 ready-to-eat foods, were herein performed. Two novel STs were identified, and a strain belonging to the alarming MRSA-ST80-SCC*mec*-IV European clone was detected. An up-to-date assessment of *se* and *sel* genes in our foodborne *S. aureus* strains was carried out, and we found that MSSA strains produced higher amounts of enterotoxins than the MRSA-ST80-SCC*mec*-IV strain herein sequenced. The detection of antimicrobial resistance genetic determinants in the 12 *S. aureus* strains supported the results of the antibiotic susceptibility testing previously performed by Mekhoulfi et al. [4]. Furthermore, a plethora of virulence genes were found in the 12 *S. aureus* genomes, although SA82 lacked several determinants involved in adhesion and colonisation, confirming its outgrouping position in the taxonomic clustering.

The results of the genomic analysis, together with the production of enterotoxins investigated in this study, confirm the risk associated with the spread of this pathogen in food, which is worsened by the observation that some of these *S. aureus* strains are multidrug resistant, and by the detection of the *tst* gene, determinant for the toxic shock syndrome toxin-1, in *S. aureus* SA82, and the PVL-encoding genes in the methicillin-resistant SA46 strain.

## 5. Materials and Methods

### 5.1. S. aureus Strains Used in the Study

The 12 *S. aureus* strains sequenced in this study are included in Supplementary Table S4. These strains were cultured on Baird–Parker egg yolk (BPEY, Oxoid, France), and their purity was checked by streaking on the same agar medium. The pure cultures were stored as stock cultures at −80° C in brain heart infusion broth (BHI; Conda. Pronadisa, Spain) supplemented with 0.6% yeast extract (Biolife Italiana, Milan, Italy) and 20% glycerol. For DNA extraction, the working culture was prepared as described by Fusco et al. [111].

### 5.2. Whole-Genome Sequencing and Analysis

Two millilitres of fresh working cultures of each of the *S. aureus* strains were used for the DNA extraction following the protocol described by Chieffi et al. [7]. The integrity, purity, and quantity of DNA were assessed by agarose gel electrophoresis, a Nanodrop photometer (Peqlab-VWR International Srl, Darmstadt, Germany), and a Qubit 3.0 fluorimeter (Thermo Fisher Scientific, Waltham, MA, USA). DNA was subjected to whole-genome shotgun sequencing by using an Illumina mate pair library prep kit (Illumina, San Diego, CA, USA), according to the manufacturer’s instructions, and then sequenced on an Illumina MiSeq platform with the 2 × 250 mate pair procedure. Reads were then trimmed by NxTrim (V2) [112], and *de novo* assembly was performed using SPAdes version 3.10.1 (©St. Petersburg State University, St. Petersburg, Russia [113]) with the following parameters: Kmer 21, 33, 55, 77 and “careful”.

The overall contiguity of the assembly and genome statistics were determined with MIGA [114]. The completeness of the *de novo* assemblies was measured by the presence of 106 single-copy genes that are observed across almost all prokaryotic genomes by using MiGA [114]. Contamination was calculated based on the copy number of essential genes (in %) present in the genome. Quality scores were then calculated as completeness percentage minus five times contamination percentage.

The whole-genome shotgun projects have been deposited at DDBJ/ENA/GenBank under the accessions reported in Supplementary Table S4. The versions described in this paper are JAHLTR010000000 for *S. aureus* SA01, JAFNJM010000000 for *S. aureus* SA02, JAFNJN010000000 for *S. aureus* SA04, JAHLTS010000000 for *S. aureus* SA07, JAHLTT010000000 for *S. aureus* SA08, JAHLTU010000000 for *S. aureus* SA10, JAFNJO010000000 for *S. aureus* SA18, JAFNJP010000000 *S. aureus* SA20, JAFRED010000000 for *S. aureus* SA24, JAFNJQ010000000 for *S. aureus* SA46, JAFNJR010000000 for *S. aureus* SA51, and JAHLTV010000000 for *S. aureus* SA82.

### 5.3. Bioinformatic Methods

#### 5.3.1. Gene Prediction, ANI, and Phylogenomic Analysis

Genes were predicted and annotated using the PROKKA pipeline implemented in the Galaxy platform (Galaxy Tool Version 1.14.5 [115]) and by NCBI (National Center for Biotechnology Information) Prokaryotic Genome Annotation Pipeline (PGAP [116]). Protein ID used in the manuscript indicated those obtained by PGAP.

All the protein sequences used in this study were retrieved from GenBank (NCBI). The homology-based relationship of *S. aureus* genes and predicted proteins towards reference sequences was determined by the BLASTN and BLASTP algorithms on the NCBI site [117]. Gene models were manually determined, and clustering and orientation were subsequently deduced for the closely linked genes.

Strains used to perform pan-genome and phylogenetic analyses are listed in Supplementary Table S4. Genomic sequences were downloaded from the NCBI and submitted to the PROKKA pipeline. The obtained .gff3 files were used as input for Roary (Galaxy Version 3.13.0 + galaxy1), the pangenome pipeline [118], to generate a core gene alignment with 95% as the minimum percentage identity for blastp analysis and 99% as the total percentage of the isolates in which the gene needs to be present for it to be considered a core gene. The pan-genome was represented as the core genome (shared by >99% of strains), accessory genome (genes present in >2 strains but not in all), and unique genome (genes unique to individual strains). The total pan-genome was also shown as core (99% ≤ strains ≤ 100%), soft core (95% ≤ strains < 99%), shell (15% ≤ strains  < 95%), and cloud (0% ≤ strains  < 15%). Visualisation of the output was achieved by using Phandango (version 1.3.0 [119]).

The genetic divergence among *S. aureus* species was calculated using the ANI calculator [120,121], which estimates the average nucleotide identity (ANI) using both best hits (one-way ANI) and reciprocal best hits (two-way ANI) between genomic datasets.

Genome-based phylogeny using 1000 single copy genes was reconstructed employing the Phylogenetic Tree Building Service implemented in the BV-BRC platform [122], with the maximum likelihood method RAxML (version 8.2.11) and progressive refinement [123]. *S. argenteus* MSHR1132 was used as an outgroup, and visualisation of the phylogenetic trees was performed by using iTOL (version 6.3.2 [124]).

#### 5.3.2. MLST and *spa* Typing

Multilocus sequence typing (MLST) was performed by MLST 2.0 (software version: 2.0.4 (8 May 2019); database version: 2.0.0 (13 September 2021)) of the Center for Genomic Epidemiology [125,126]. *spa* typing was performed by spaTyper (Software version 1.0 Database version: 22 August 2022) of the Center for Genomic Epidemiology [127,128].

#### 5.3.3. Antibiotic Resistance and Virulence Determinants Analyses

Antibiotic resistance determinants were computationally predicted within the BV-BRC platform [129] by a k-mer-based detection method and BLAST analysis, and then manually curated. Virulence determinants were screened by using the comparative pathogenomics-based VF analysis pipeline VFanalyzer [130] implemented in the virulence factor database (VFDB) [131], and then manually curated. A heatmap was manually constructed and visualised by using the heatmapper web server [43,132] with average linkage as the clustering method and Euclidean distance measurement method.

Enterotoxin genes were determined by homology search using previously reported reference sequences ([1]; Supplementary Table S7) using an E-value cut-off of 0.001, a minimum percentage of alignment identity of 80%, and a relative coverage threshold of >80% [41,133].

Prediction of SCC*mec* elements in sequenced *S. aureus* isolates was performed by using the SCC*mec*Finder v1.2 [134,135] with default parameters, then manually curated.

### 5.4. Cultivation of S. aureus for Staphylococcal Enterotoxin Production Assessment

*S. aureus* strains SA04, SA18, SA20, SA46, and SA51, harbouring the classical and some newly described *se* genes (namely *sea*, *seb*, *sed*, *seh*, and *ser*), were chosen to assess the ability to produce SEs. Bacterial cultures were prepared as described by Schubert et al. [136]. Briefly, 100 mL of brain heart infusion supplemented with 1% yeast extract (BHI + YE, Biocorp, Warsaw, Poland) were inoculated with pre-culture to reach an optical density of 0.02 at 600 nm (OD_600_). Prior to inoculation, the pre-cultures were washed twice with phosphate-buffered saline (PBS) to remove residual BHI broth and enterotoxins (repeated centrifugation at 12,000× *g* for 5 min and resuspension in PBS). Cultures were incubated at 37 °C with constant agitation at 230 rpm. The bacterial cell concentration of the broth cultures at 0, 24, and 48 h of incubation was determined by plating serial tenfold dilutions, prepared in quarter-strength Ringer’s solution (Oxoid, Basingstoke, UK), onto BHI agar. The pH of the broth cultures was measured using a FE20-FiveEasy™ pH-meter (Mettler-Toledo, Greifensee, Switzerland).

### 5.5. Sandwich ELISA for SEA, SEB, SED, SEH, and SER Detection

Recombinant staphylococcal enterotoxins (rSED, rSEH, and rSER) were obtained as previously described by Schubert et al. [136,137]. Rabbit polyclonal anti-A, anti-B, anti-SED, and anti-SER antibodies were purchased from OriGene Technologies GmbH (Herford, Germany), whereas rabbit polyclonal anti-H and anti-H(HRP) antibodies were purchased from Abcam (Cambridge, UK). Samples for staphylococcal enterotoxins (SEs) detection were collected after 24 and 48 h of growth and stored at −20 °C until analysed. Supernatants were pre-incubated with a 20% normal rabbit serum to bind protein A and then diluted in PBS containing 0.1% Tween-20 (Merck, Darmstadt, Germany). The enzyme linked immunosorbent assay (ELISA) was also performed as described by Schubert et al. [136,137]. The concentration of the SEs in samples was measured with SEA, SEB (Merck KGaA, Darmstadt, Germany), rSED, rSEH, and rSER as standards, using a 4-parameter logistic curve fit. The ELISA assays for each SE were run in two biological repeats with two replicates. Data analysis was carried out using GraphPad Prism software 8.0.1 (GraphPad Software Inc., La Jolla, CA, USA).

### 5.6. RNA Extraction and Reverse Transcription–Quantitative PCR to Assess seh Gene Expression

Expression of the *seh* gene was assessed in the *seh*-positive *S. aureus* SA04 and SA46 strains. Samples for RNA isolation were collected from the BHI+YE broth after 5 and 24 h of growth. RNA extraction, purification, cDNA synthesis, and reverse transcription–quantitative polymerase chain reaction (RT-qPCR) were performed as described by Schubert et al. [136]. The *rpo*B housekeeping gene was used for normalisation [138]. Transcript levels of *seh* relative to *rpo*B were calculated according to Pfaffl [139]. Each RT-qPCR assay consisted of two biological repeats with three replicates. Data analysis was carried out using Bio-Rad CFX Manager software.

## Figures and Tables

**Table 1:** Main genetic features of the sequenced *S. aureus* strains isolated from Algerian ready-to-eat foods.

Strain	MSSA or MRSA	Origin	ST	*se* and *sel* Genes	*mec*A (SCC*mec*-Type)	*tst*	*luk*F-PV and *luk*S-PV
SA01	MSSA	Potato in sauce	15	*selw*^a^, *selx*^h^	-	-	-
SA02	MSSA	Cooked meat	5	*egc*1 (*seg*, *sei*, *sem*, *sen*, *seo*, *ψent*1-*ψent*2 *), *selw* *^,b^, *selx*1	-	-	-
SA04	MSSA	Cooked meat	1	*sea*1, *seh*, *sek*, *seq*, *selw*^a^, *selx*5	-	-	-
SA07	MSSA	Cooked meat	7199 ^k^	*selw*^a^, *selx*^h^	-	-	-
SA08	MSSA	Cooked meat	25	*egc*5 (*seg*, *sei*, *sem*, *sen*, *seo*, *selu*2), *selw*^d^, *selx*9 ^i^	-	-	-
SA10	MSSA	Cooked meat	101	*selw*^e^, *selx*^h^	-	-	-
SA18	MSSA	Pizza	97	*seb*v1, *selw* *^,f^, *selx*bov2	-	-	-
SA20	MSSA	Cooked meat	8	*sed* *^,j^, *selj*, *ser*, *selw* ^c^, *selx*2	-	-	-
SA24	MSSA	Lentil soup	7200 ^k^	*egc*5 (*seg*, *sei*, *sem*, *sen*, *seo*, *selu*2), *selw* *^,f^, *selx*5	-	-	-
SA46	MRSA	Braised beef	80	*seh* *, *selw* ^g^, *selx* ^h^, *sey*	+(SCC*mec*-IV)	-	+
SA51	MSSA	Fermented milk	5	*sea*2 ^i^, *egc*1 (*seg*, *sei*, *sem*, *sen*, *seo*, *ψent*1-*ψent*2 *), *selw* *^,b^, *selx*1, *sel*30	-	-	-
SA82	MSSA	Sautéed beef with potato	22	*egc*5 (*seg*, *sei*, *sem*, *sen*, *seo*, *selu*2), *selw*^d^, *selx*6	-	+	-

* pseudogene; + present; - absent; ^a^ *selw* belonging to group 3 [14]; ^b^ Nonfunctional *selw* belonging to group 4 [14]; ^c^ *selw* belonging to group 2 [14]; ^d^ 98% similarity to *selw* genes belonging to group 1 [14]; ^e^ 98% similarity to *selw* gene belonging to group 2 [14]; ^f^ 98% similarity to nonfunctional *selw* gene belonging to group 4 [14]; ^g^ 99% similar to *selw* gene belonging to group 6 [14]; ^h^ Different allelic variant than those previously described for *selx* [7,15,16]; ^i^ 1 nucleotide substitution compared with the described allelic variant (not leading to amino acid sequence variation in the predicted protein) [15,17]; ^j^ Different allelic variant than the truncated *sed*v3 described by Johler et al. [18]; ^k^ Novel ST.

**Table 2:** Antimicrobial resistance profiles and related genetic determinants of *S. aureus* strains isolated from Algerian ready-to-eat foods.

Strain	Resistance Profile ^a^	Genetic Determinants
SA01	P	*bla*Z
SA02	OF, ERY, L, TE	*gyr*A-S84L, *erm*B, *lmr*S, *tet*(L), *tet*(38)
SA04	P	*bla*Z
SA07	P	*bla*Z
SA08	P	*bla*Z
SA10	P	*bla*Z
SA18	P, KAN, TE	*bla*Z, *aph*(3′)-IIIa, *ant*(6)-I, *tet*(L), *tet*(38)
SA20	P	*bla*Z
SA24	P	*bla*Z
SA46	P, OXA, FOX, KAN	*mec*A, *aph*(3′)-IIIa, *ant*(6)-I
SA51	- ^b^	*bla*Z
SA82	P	*bla*Z

P: Benzylpenicillin; OF: ofloxacin; ERY: erythromycin; L: lincomycin; KAN: kanamycin; TE: tetracycline; OXA: oxacillin; FOX: cefoxitin; ^a^ As reported by Mekhloufi et al. [4]; ^b^ Sensitive to all tested antibiotics (benzylpenicillin, oxacillin, cefoxitin, gentamicin, kanamycin, tobramycin, ofloxacin, erythromycin, lincomycin, clindamycin, pristinamycin, linezolid, teicoplanin, vancomycin, tetracycline, fosfomycin, nitrofurantoin, fusidic acid, rifampicin, and co-trimoxazole) [4].

**Table 3:** Growth and pH values of *S. aureus* cultures in BHI + YE broth.

Strain	0 h		24 h		48 h	
	Log cfu/mL	pH	Log cfu/mL	pH	Log cfu/mL	pH
SA04	6.77 ± 0.58	7.61	9.22 ± 0.03	6.86	9.22 ± 0.12	7.53
SA18	6.91 ± 0.07	7.61	9.34 ± 0.23	6.97	9.44 ± 0.28	7.57
SA20	6.91 ± 0.29	7.61	8.97 ± 0.09	6.71	9.08 ± 0.13	7.29
SA46	6.39 ± 0.26	7.61	9.29 ± 0.08	7.05	9.26 ± 0.08	7.61
SA51	6.38 ± 0.28	7.61	9.15 ± 0.11	6.81	9.16 ± 0.07	7.23

**Table 4:** Production of SEA, SEB, SED, SEH, and SER (ng/mL) by *S. aureus* strains in BHI + YE broth.

Strain	SEA		SEB		SED		SEH		SER	
	24 h	48 h	24 h	48 h	24 h	48 h	24 h	48 h	24 h	48 h
SA04	156.04 ± 36.58	345.02 ± 62.67	-	-	-	-	221.76 ± 38.15	239.48 ± 84.83	-	-
SA18	-	-	2871.28 ± 811.09	14,739.17 ± 5077.70	-	-	-	-	-	-
SA20	-	-	-	-	322.70 ± 41.17	398.94 ± 64.79	-	-	36,720.10 ± 6272.32	63,176.06 ± 14,422.93
SA46	-	-	-	-	-	-	1.76 ± 2.26	N.D.	-	-
SA51	64.54 ± 9.29	74.91 ± 7.36	-	-	-	-	-	-	-	-

N.D.: not detectable.

## Data Availability

Data is contained within the article or Supplementary Materials.

## Supplementary Materials

The following supporting information can be downloaded at: https://www.mdpi.com/article/10.3390/toxins14110731/s1, Supplementary Figure S1. Pan-genome analysis of *S. aureus* strains. Visualisation of pan-genome analysis of *S. aureus* strains was performed by using Phandango (version 1.3.0). Supplementary Figure S2. Enterotoxin gene cluster (*egc*) localisation in *S. aureus* strains. Protein IDs are indicated in the figure. Red: enterotoxins; yellow: serine proteases; brown: leukocidins; grey: hypothetical proteins; light blue: restriction endonucleases; pink: transposases; turquoise: lantibiotic cluster; *cap*B: calcium binding protein; *pep*A1: glutamyl aminopeptidase; * indicates a pseudogene. Supplementary Table S1. Genomic features of *S. aureus* strains sequenced in this study. Supplementary Table S2. MLST allelic profile of sequenced *S. aureus* strains. Supplementary Table S3. *spa* typing of *S. aureus* strains. Supplementary Table S4. List of *S. aureus* strains used in this study. Supplementary Table S5. ANI values of *S. aureus* strains. Supplementary Table S6. Roary statistics. Supplementary Table S7. *se* and *sel* genes and predicted protein sequences in *S. aureus* strains. Supplementary Table S8. Virulence determinants in *S. aureus* strains.

## References

[1] Dicks J., Turnbull J.D., Russell J., Parkhill J., Alexander S. (2021). Genome sequencing of a historic *Staphylococcus aureus* collection reveals new enterotoxin genes and sheds light on the evolution and genomic organization of this key virulence gene family. J. Bacteriol..

[2] Foster T.J. (2017). Antibiotic resistance in *Staphylococcus aureus*. Current status and future prospects. FEMS Microbiol. Rev..

[3] Fusco V., Chieffi D., Fanelli F., Logrieco A.F., Cho G.-S., Kabisch J., Böhnlein C., Franz C.M.A.P. (2020). Microbial quality and safety of milk and milk products in the 21st century. Compr. Rev. Food Sci. Food Saf..

[4] Mekhloufi O.A., Chieffi D., Hammoudi A., Bensefia S.A., Fanelli F., Fusco V. (2021). Prevalence, enterotoxigenic potential and antimicrobial resistance of *Staphylococcus aureus* and Methicillin-Resistant *Staphylococcus aureus* (MRSA) isolated from Algerian ready to eat foods. Toxins.

[5] Fishovitz J., Hermoso J.A., Chang M., Mobashery S. (2014). Penicillin-binding protein 2a of methicillin-resistant *Staphylococcus aureus*. IUBMB Life.

[6] Jarraud S., Peyrat M.A., Lim A., Tristan A., Bes M., Mougel C., Etienne J., Vandenesch F., Bonneville M., Lina G. (2001). egc, a highly prevalent operon of enterotoxin gene, forms a putative nursery of superantigens in *Staphylococcus aureus*. J. Immunol..

[7] Chieffi D., Fanelli F., Cho G.-S., Schubert J., Blaiotta G., Franz C.M.A.P., Bania J., Fusco V. (2020). Novel insights into the enterotoxigenic potential and genomic background of *Staphylococcus aureus* isolated from raw milk. Food Microbiol..

[8] Achek R., El-Adawy H., Hotzel H., Hendam A., Tomaso H., Ehricht R., Neubauer H., Nabi I., Hamdi T.M., Monecke S. (2021). Molecular Characterization of *Staphylococcus aureus* Isolated from Human and Food Samples in Northern Algeria. Pathogens.

[9] Chaalal W., Chaalal N., Bourafa N., Kihal M., Diene S.M., Rolain J.-M. (2018). Characterization of *Staphylococcus aureus* Isolated from Food Products in Western Algeria. Foodborne Pathog. Dis..

[10] Titouche Y., Hakem A., Houali K., Meheut T., Vingadassalon N., Ruiz-Ripa L., Salmi D., Chergui A., Chenouf N., Hennekinne J.A. (2019). Emergence of methicillin-resistant *Staphylococcus aureus* (MRSA) ST8 in raw milk and traditional dairy products in the Tizi Ouzou area of Algeria. J. Dairy Sci..

[11] Titouche Y., Houali K., Ruiz-Ripa L., Vingadassalon N., Nia Y., Fatihi A., Cauquil A., Bouchez P., Bouhier L., Torres C. (2020). Enterotoxin genes and antimicrobial resistance in *Staphylococcus aureus* isolated from food products in Algeria. J. Appl. Microbiol..

[12] Breurec S., Fall C., Pouillot R., Boisier P., Brisse S., Diene-Sarr F., Djibo S., Etienne J., Fonkoua M.C., Perrier-Gros-Claude J.D. (2011). Epidemiology of methicillin-susceptible *Staphylococcus aureus* lineages in five major African towns: High prevalence of Panton-Valentine leukocidin genes. Clin. Microbiol. Infect..

[13] Lozano C., Gharsa H., Ben Slama K., Zarazaga M., Torres C. (2016). *Staphylococcus aureus* in animals and food: Methicillin resistance, prevalence and population structure. A review in the African continent. Microorganisms.

[14] Aung M., San T., Aye M., Mya S., Maw W., Zan K., Htut W., Kawaguchiya M., Urushibara N., Kobayashi N. (2017). Prevalence and Genetic Characteristics of *Staphylococcus aureus* and *Staphylococcus argenteus* Isolates Harboring Panton-Valentine Leukocidin, Enterotoxins, and TSST-1 Genes from Food Handlers in Myanmar. Toxins.

[15] Wilson G.J., Seo K.S., Cartwright R.A., Connelley T., Chuang-Smith O.N., Merriman J.A., Guinane C.M., Park J.Y., Bohach G.A., Schlievert P.M. (2011). A novel core genome-encoded superantigen contributes to lethality of community-associated MRSA necrotizing pneumonia. PLoS Pathog..

[16] Roetzer A., Haller G., Beyerly J., Geier C.B., Wolf H.M., Gruener C.S., Model N., Eibl M.M. (2016). Genotypic and phenotypic analysis of clinical isolates of *Staphylococcus aureus* revealed production patterns and hemolytic potentials unlinked to gene profiles and source. BMC Microbiol..

[17] Borst D.W., Betley M.J. (1994). Phage-associated differences in staphylococcal enterotoxin A gene (*sea*) expression correlate with sea allele class. Infect. Immun..

[18] Johler S., Sihto H.-M., Macori G., Stephan R. (2016). Sequence variability in staphylococcal enterotoxin genes *seb*, *sec*, and *sed*. Toxins.

[19] Baba T., Takeuchi F., Kuroda M., Yuzawa H., Aoki K., Oguchi A., Nagai Y., Iwama N., Asano K., Naimi T. (2002). Genome and virulence determinants of high virulence community-acquired MRSA. Lancet.

[20] Betley M.J., Mekalanos J.J. (1988). Nucleotide sequence of the type A staphylococcal enterotoxin gene. J. Bacteriol..

[21] Chua K., Seemann T., Harrison P.F., Davies J.K., Coutts S.J., Chen H., Haring V., Moore R., Howden B.P., Stinear T.P. (2010). Complete Genome Sequence of *Staphylococcus aureus* Strain JKD6159, a Unique Australian Clone of ST93-IV Community Methicillin-Resistant *Staphylococcus aureus*. J. Bacteriol..

[22] Collery M.M., Smyth C.J. (2007). Rapid differentiation of *Staphylococcus aureus* isolates harbouring *egc* loci with pseudogenes psient1 and psient2 and the selu or seluv gene using PCR-RFLP. J. Med. Microbiol..

[23] Egyir B., Hadjirin N.F., Gupta S., Owusu F., Agbodzi B., Adogla-Bessa T., Addo K.K., Stegger M., Larsen A.R., Holmes M.A. (2020). Whole-genome sequence profiling of antibiotic-resistant *Staphylococcus aureus* isolates from livestock and farm attendants in Ghana. J. Glob. Antimicrob. Resist..

[24] Gawlik D., Ruppelt-Lorz A., Muller E., Reißig A., Hotzel H., Braun S.D., Söderquist B., Ziegler-Cordts A., Stein C., Pletz M.W. (2020). Molecular investigations on a chimeric strain of *Staphylococcus aureus* sequence type 80. PLoS ONE.

[25] Jørgensen H.J., Mathisen T., Lovseth A., Omoe K., Qvale K.S., Loncarevic S. (2005). An outbreak of staphylococcal food poisoning caused by enterotoxin H in mashed potato made with raw milk. FEMS Microbiol. Lett..

[26] Langley R.J., Ting Y.T., Clow F., Young P.G., Radcliff F.J., Choi J.M., Sequeira R.P., Holtfreter S., Baker H., Fraser J.D. (2017). Staphylococcal enterotoxin-like X (SElX) is a unique superantigen with functional features of two major families of staphylococcal virulence factors. PLoS Pathog..

[27] Sabirova J.S., Xavier B.B., Hernalsteens J.P., De Greve H., Ieven M., Goossens H., Malhotra-Kumar S. (2014). Complete Genome Sequences of Two Prolific Biofilm-Forming *Staphylococcus aureus* Isolates Belonging to USA300 and EMRSA-15 Clonal Lineages. Genome Announc..

[28] Thomas D.Y., Jarraud S., Lemercier B., Cozon G., Echasserieau K., Etienne J., Gougeon M.L., Lina G., Vandenesch F. (2006). Staphylococcal enterotoxin-like toxins U2 and V, two new staphylococcal superantigens arising from recombination within the enterotoxin gene cluster. Infect. Immun..

[29] Tuffs S.W., James D.B.A., Bestebroer J., Richards A.C., Goncheva M.I., O’Shea M., Wee B.A., Seo K.S., Schlievert P.M., Lengeling A. (2017). The *Staphylococcus aureus* superantigen SElX is a bifunctional toxin that inhibits neutrophil function. PLoS Pathog..

[30] Utter B., Deutsch D.R., Schuch R., Winer B.Y., Verratti K., Bishop-Lilly K., Sozhamannan S., Fischetti V.A. (2014). Beyond the Chromosome: The Prevalence of Unique Extra-Chromosomal Bacteriophages with Integrated Virulence Genes in Pathogenic *Staphylococcus aureus*. PLoS ONE..

[31] Wan T.W., Liu Y.J., Wang Y.T., Lin Y.T., Hsu J.C., Tsai J.C., Chiu H.C., Hsueh P.R., Hung W.C., Teng L.J. (2022). Potentially conjugative plasmids harboring Tn6636, a multidrug-resistant and composite mobile element, in *Staphylococcus aureus*. J. Microbiol. Immunol. Infect..

[32] Okumura K., Shimomura Y., Murayama S.Y., Yagi J., Ubukata K., Kirikae T., Miyoshi-Akiyama T. (2012). Evolutionary paths of streptococcal and staphylococcal superantigens. BMC Genom..

[33] Kläui A.J., Boss R., Graber H.U. (2019). Characterization and comparative analysis of the *Staphylococcus aureus* genomic island *v*Saβ: An in silico Approach. J. Bacteriol..

[34] Higgins K.A., Peng H., Luebke J.L., Chang F.M., Giedroc D.P. (2015). Conformational analysis and chemical reactivity of the multidomain sulfurtransferase, *Staphylococcus aureus* CstA. Biochemistry.

[35] Sato’o Y., Omoe K., Ono H.K., Nakane A., Hu D.L. (2013). A novel comprehensive analysis method for *Staphylococcus aureus* pathogenicity islands. Microbiol. Immunol..

[36] Shearer J.E., Wireman J., Hostetler J., Forberger H., Borman J., Gill J., Sanchez S., Mankin A., Lamarre J., Lindsay J.A. (2011). Major families of multiresistant plasmids from geographically and epidemiologically diverse staphylococci. G3 Genes Genomes Genet..

[37] Rowland S.J., Dyke K.G. (1989). Characterization of the staphylococcal beta-lactamase transposon Tn552. EMBO J..

[38] Rowland S.J., Dyke K.G. (1990). Tn552, a novel transposable element from *Staphylococcus aureus*. Mol Microbiol..

[39] Jensen S.O., Lyon B.R. (2009). Genetics of antimicrobial resistance in *Staphylococcus aureus*. Future Microbiol..

[40] Peschel A., Otto M. (2013). Phenol-soluble modulins and staphylococcal infection. Nat. Rev. Microbiol..

[41] Gordon N.C., Price J.R., Cole K., Everitt R., Morgan M., Finney J., Kearns A.M., Pichon B., Young B., Wilson D.J. (2014). Prediction of *Staphylococcus aureus* antimicrobial resistance by whole-genome sequencing. J. Clin. Microbiol..

[42] Dumitrescu O., Dauwalder O., Boisset S., Reverdy M.É., Tristan A., Vandenesch F. (2010). *Staphylococcus aureus* resistance to antibiotics: Key points in 2010. Med. Sci..

[43] Heatmapper. http://www.heatmapper.ca.

[44] McCarthy A.J., Lindsay J.A. (2013). *Staphylococcus aureus* innate immune evasion is lineage-specific: A bioinfomatics study. Infect. Genet. Evol..

[45] Aswani V., Najar F., Pantrangi M., Mau B., Schwan W.R., Shukla S.K. (2019). Virulence factor landscape of a *Staphylococcus aureus* sequence type 45 strain, MCRF184. BMC Genom..

[46] Gravet A., Colin D.A., Keller D., Girardot R., Monteil H., Prévost G. (1998). Characterization of a novel structural member, LukE-LukD, of the bi-component staphylococcal leucotoxins family. FEBS Lett..

[47] Rhem M.N., Lech E.M., Patti J.M., McDevitt D., Höök M., Jones D.B., Wilhelmus K.R. (2000). The collagen-binding adhesin is a virulence factor in *Staphylococcus aureus* keratitis. Infect. Immun..

[48] Agabou A., Ouchenane Z., Ngba Essebe C., Khemissi S., Chehboub M.T.E., Chehboub I.B., Sotto A., Dunyach-Remy C., Lavigne J.P. (2017). Emergence of nasal carriage of ST80 and ST152 PVL+ *Staphylococcus aureus* isolates from livestock in Algeria. Toxins.

[49] Mairi A., Touati A., Pantel A., Zenati K., Martinez A.Y., Dunyach-Remy C., Sotto A., Lavigne J.P. (2019). Distribution of toxinogenic Methicillin-Resistant and Methicillin-Susceptible *Staphylococcus aureus* from different ecological niches in Algeria. Toxins.

[50] Ghebremedhin B., Olugbosi M.O., Raji A.M., Layer F., Bakare R.A., König B., König W. (2009). Emergence of a community-associated methicillin-resistant *Staphylococcus aureus* strain with a unique resistance profile in Southwest Nigeria. J. Clin. Microbiol..

[51] Schaumburg F., Ngoa U.A., Kösters K., Köck R., Adegnika A.A., Kremsner P.G., Lell B., Peters G., Mellmann A., Becker K. (2011). Virulence factors and genotypes of *Staphylococcus aureus* from infection and carriage in Gabon. Clin. Microbiol. Infect..

[52] Lakhundi S., Zhang K. (2018). Methicillin-Resistant *Staphylococcus aureus*: Molecular Characterization, Evolution, and Epidemiology. Clin. Microbiol. Rev..

[53] Thapaliya D., Forshey B.M., Kadariya J., Quick M.K., Farina S., O’ Brien A., Nair R., Nworie A., Hanson B., Kates A. (2017). Prevalence and molecular characterization of *Staphylococcus aureus* in commercially available meat over a one-year period in Iowa, USA. Food Microbiol..

[54] Mama O.M., Morales L., Ruiz-Ripa L., Zarazaga M., Torres C. (2020). High prevalence of multidrug resistant *S. aureus*-CC398 and frequent detection of enterotoxin genes among non-CC398 *S. aureus* from pig-derived food in Spain. Int. J. Food Microbiol..

[55] Casman E.P., Bennett R.W., Dorsey A.E., Issa J.A. (1967). Identification of a fourth staphylococcal enterotoxin, enterotoxin D. J. Bacteriol..

[56] Bergdoll M.S., Borja C.R., Robbins R.N., Weiss K.F. (1971). Identification of enterotoxin E. Infect Immun..

[57] Benkerroum N. (2018). Staphylococcal enterotoxins and enterotoxin-like toxins with special reference to dairy products: An overview. Crit. Rev. Food Sci. Nutr..

[58] Ikeda T., Tamate N., Yamaguchi K., Makino S. (2005). Mass outbreak of food poisoning disease caused by small amounts of staphylococcal enterotoxins A and H. Appl. Environ. Microbiol..

[59] Johler S., Giannini P., Jermini M., Hummerjohann J., Baumgartner A., Stephan R. (2015). Further evidence for staphylococcal food poisoning outbreaks caused by *egc*-encoded enterotoxins. Toxins.

[60] Suzuki Y., Kobayashi M., Matsushita S., Uehara S., Kato R., Sato’o Y., Ono H.K., Sadamasu K., Kai A., Kamata Y. (2015). Detection of the staphylococcal enterotoxin D-like gene from staphylococcal food poisoning isolates over the last two decades in Tokyo. J. Vet. Med. Sci..

[61] Umeda K., Nakamura H., Yamamoto K., Nishina N., Yasufuku K., Hirai Y., Hirayama T., Goto K., Hase A., Ogasawara J. (2017). Molecular and epidemiological characterization of staphylococcal foodborne outbreak of *Staphylococcus aureus* harboring *seg, sei, sem, sen, seo,* and *selu* genes without production of classical enterotoxins. Int. J. Food Microbiol..

[62] Denayer S., Delbrassinne L., Nia Y., Botteldoorn N. (2017). Food-Borne Outbreak investigation and molecular typing: High diversity of *Staphylococcus aureus* strains and importance of toxin detection. Toxins.

[63] Johler S., Weder D., Bridy C., Huguenin M.-C., Robert L., Hummerjohann J., Stephan R. (2015). Outbreak of staphylococcal food poisoning among children and staff at a Swiss boarding school due to soft cheese made from raw milk. J. Dairy Sci..

[64] Asao T., Kumeda Y., Kawai T., Shibata T., Oda H., Haruki K., Nakazawa H., Kozaki S. (2003). An extensive outbreak of staphylococcal food poisoning due to low-fat milk in Japan: Estimation of enterotoxin A in the incriminated milk and powdered skim milk. Epidemiol. Infect..

[65] Evenson M.L., Ward Hinds M., Bernstein R.S., Bergdoll M.S. (1988). Estimation of human dose of staphylococcal enterotoxin A from a large outbreak of staphylococcal food poisoning involving chocolate milk. Int. J. Food Microbiol..

[66] Harbrecht D.F., Bergdoll M. (1980). Staphylococcal enterotoxin B production in hard-boiled eggs. J. Food Sci..

[67] Guidi F., Duranti A., Gallina S., Nia Y., Petruzzelli A., Romano A., Travaglini V., Olivastri A., Calvaresi V., Decastelli L. (2018). Characterization of A Staphylococcal Food Poisoning Outbreak in A Workplace Canteen during the Post-Earthquake Reconstruction of Central Italy. Toxins.

[68] Lis E., Podkowik M., Schubert J., Bystroń J., Stefaniak T., Bania J. (2012). Production of Staphylococcal Enterotoxin R by *Staphylococcus aureus* Strains. Foodborne Pathog. Dis..

[69] Hait J.M., Bennett R.W., Monday S.R. (2018). Staphylococcal enterotoxin type r pseudogene presence in *Staphylococcus aureus* reference and outbreak strains. J. AOAC Int..

[70] Lis E., Podkowik M., Bystroń J., Stefaniak T., Bania J. (2012). Temporal expression of staphylococcal enterotoxin H in comparison with accessory gene regulator–dependent and –independent enterotoxins. J. Food Prot..

[71] Jones T.F., Kellum M.E., Porter S.S., Bell M., Schaffner W. (2002). An outbreak of community-acquired foodborne illness caused by methicillin-resistant *Staphylococcus aureus*. Emerg. Infect. Dis..

[72] Peeters L.E.J., Argudín M.A., Azadikhah S., Butaye P. (2015). Antimicrobial resistance and population structure of *Staphylococcus aureus* recovered from pigs farms. Vet. Microbiol..

[73] Aung K.T., Hsu L.Y., Koh T.H., Hapuarachchi H.C., Chau M.L., Gutiérrez R.A., Ng L.C. (2017). Prevalence of methicillin-resistant *Staphylococcus aureus* (MRSA) in retail food in Singapore. Antimicrob. Resist. Infect. Control..

[74] Zhang C., Shen Y., Dong M. (2013). Distribution, polymorphism and temporal expression of *egc* in *Staphylococcus aureus* isolates from various foods in China. Food Control.

[75] Fischer A.J., Kilgore S.H., Singh S.B., Allen P.D., Hansen A.R., Limoli D.H., Schlievert P.M. (2019). High prevalence of *Staphylococcus aureus* enterotoxin gene cluster superantigens in cystic fibrosis clinical isolates. Genes.

[76] Grema H.A., Geidam Y.A., Gadzama G.B., Ameh J.A., Suleiman A. (2015). Methicillin resistant *Staphylococcus aureus* (MRSA): A review. Adv. Anim. Vet. Sci..

[77] Pekana A., Green E. (2018). Antimicrobial resistance profiles of *Staphylococcus aureus* isolated from meat carcasses and bovine milk in abattoirs and dairy farms of the Eastern Cape, South Africa. Int. J. Environ. Res. Public Health.

[78] Okorie-Kanu O.J., Anyanwu M.U., Ezenduka E.V., Mgbeahuruike A.C., Thapaliya D., Gerbig G., Ugwuijem E.E., Okorie-Kanu C.O., Agbowo P., Olorunleke S. (2020). Molecular epidemiology, genetic diversity and antimicrobial resistance of *Staphylococcus aureus* isolated from chicken and pig carcasses, and carcass handlers. PLoS ONE.

[79] Sadat A., Shata R.R., Farag A.M.M., Ramadan H., Alkhedaide A., Soliman M.M., Elbadawy M., Abugomaa A., Awad A. (2022). Prevalence and characterization of PVL-positive *Staphylococcus aureus* isolated from raw cow’s milk. Toxins.

[80] Chao G., Zhang X., Zhang X., Huang Y., Xu L., Zhou L., Yang W., Jiang Y., Xue F., Wu Y. (2013). Phenotypic and genotypic characterization of methicillin-resistant *Staphylococcus aureus* (MRSA) and methicillin-susceptible *Staphylococcus aureus* (MSSA) from different sources in China. Foodborne Pathog. Dis..

[81] Ge B., Mukherjee S., Hsu C.H., Davis J.A., Tran T.T.T., Yang Q., Abbott J.W., Ayers S.L., Young S.R., Crarey E.T. (2017). MRSA and multidrug-resistant *Staphylococcus aureus* in U.S. retail meats, 2010–2011. Food Microbiol..

[82] Drougka E., Foka A., Giormezis N., Sergelidis D., Militsopoulou M., Jelastopulu E., Komodromos D., Sarrou S., Anastassiou E.D., Petinaki E. (2019). Multidrug-resistant enterotoxigenic *Staphylococcus aureus* lineages isolated from animals, their carcasses, the personnel, and the environment of an abattoir in Greece. J. Food Process. Preserv..

[83] Clinical and Laboratory Standards Institute (2012). Performance Standards for Antimicrobial Susceptibility Testing.

[84] Pereira L.A., Harnett G.B., Hodge M.M., Cattell J.A., Speers D.J. (2014). Real-time PCR assay for detection of *bla*Z genes in *Staphylococcus aureus* clinical isolates. J. Clin. Microbiol..

[85] Richter S.S., Doern G.V., Heilmann K.P., Miner S., Tendolkar S., Riahi F., Diekema D.J. (2016). Detection and prevalence of penicillin-susceptible *Staphylococcus aureus* in the United States in 2013. J. Clin. Microbiol..

[86] Monecke S., Berger-Bächi B., Coombs G., Holmes A., Kay I., Kearns A., Linde H.-J., O’Brien F., Slickers P., Ehricht R. (2007). Comparative genomics and DNA array-based genotyping of pandemic *Staphylococcus aureus* strains encoding Panton-Valentine leukocidin. Clin. Microbiol. Infect..

[87] Arêde P., Ministro J., Oliveira D.C. (2013). Redefining the role of the β-lactamase locus in methicillin-resistant *Staphylococcus aureus*: β-lactamase regulators disrupt the *MecI*-mediated strong repression on *mecA* and optimize the phenotypic expression of resistance in strains with constitutive *mecA* expression. Antimicrob. Agents Chemother..

[88] Wysocka M., Monteiro T., de Pina C., Gonçalves D., de Pina S., Ludgero-Correia A., Moreno J., Zamudio R., Almebairik N., Gray L.J. (2021). Whole-genome analysis uncovers loss of *bla*Z associated with carriage isolates belonging to methicillin-resistant *Staphylococcus aureus* (MRSA) clone ST5-VI in Cape Verde. J. Glob. Antimicrob. Resist..

[89] Sabat A.J., Pournaras S., Akkerboom V., Tsakris A., Grundmann H., Friedrich A.W. (2015). Whole-genome analysis of an oxacillin-susceptible CC80 mecA-positive *Staphylococcus aureus* clinical isolate: Insights into the mechanisms of cryptic methicillin resistance. J. Antimicrob. Chemother..

[90] Mairi A., Touati A., Lavigne J.P. (2020). Methicillin-resistant *Staphylococcus aureus* ST80 clone: A systematic review. Toxins.

[91] Li G., Wu C., Wang X., Meng J. (2015). Prevalence and characterization of methicillin susceptible *Staphylococcus aureus* ST398 isolates from retail foods. Int. J. Food Microbiol..

[92] Naorem R.S., Blom J., Fekete C. (2021). Genome-wide comparison of four MRSA clinical isolates from Germany and Hungary. PeerJ.

[93] Bruce S.A., Smith J.T., Mydosh J.L., Ball J., Needle D.B., Gibson R., Andam C.P. (2022). Shared antibiotic resistance and virulence genes in *Staphylococcus aureus* from diverse animal hosts. Sci. Rep..

[94] Muneeb K.H., Sudha S., Sivaraman G.K., Ojha R., Mendem S.K., Murugesan D., Raisen C.L., Shome B., Holmes M. (2022). Whole-genome sequence analysis of *Staphylococcus aureus* from retail fish acknowledged the incidence of highly virulent ST672-MRSA-IVa/t1309, an emerging Indian clone, in Assam, India. Environ. Microbiol. Rep..

[95] Chairat S., Gharsa H., Lozano C., Gómez-Sanz E., Gómez P., Zarazaga M., Boudabous A., Torres C., Ben Slama K. (2015). Characterization of *Staphylococcus aureus* from raw meat samples in Tunisia: Detection of clonal lineage ST398 from the African continent. Foodborne Pathog. Dis..

[96] Omwenga I., Aboge G.O., Mitema E.S., Obiero G., Ngaywa C., Ngwili N., Wamwere G., Wainaina M., Bett B. (2021). Antimicrobial usage and detection of multidrug-resistant *Staphylococcus aureus*, including methicillin-resistant strains in raw milk of livestock from Northern Kenya. Microb. Drug Resist..

[97] Amoako D.G., Somboro A.M., Abia A.L.K., Allam M., Ismail A., Bester L., Essack S.Y. (2019). Genomic analysis of methicillin-resistant *Staphylococcus aureus* isolated from poultry and occupational farm workers in Umgungundlovu District, South Africa. Sci. Total Environ..

[98] Heilmann C., Hartleib J., Hussain M.S., Peters G. (2005). The multifunctional *Staphylococcus aureus* autolysin aaa mediates adherence to immobilized fibrinogen and fibronectin. Infect. Immun..

[99] Menzies B.E. (2003). The role of fibronectin binding proteins in the pathogenesis of *Staphylococcus aureus* infections. Curr. Opin. Infect. Dis..

[100] Bjerketorp J., Jacobsson K., Frykberg L. (2004). The von Willebrand factor-binding protein (vWbp) of *Staphylococcus aureus* is a coagulase. FEMS Microbiol Lett..

[101] Dinges M.M., Orwin P.M., Schlievert P.M. (2000). Exotoxins of *Staphylococcus aureus*. Clin. Microbiol. Rev..

[102] Rohmer C., Wolz C. (2021). The role of hlb-converting bacteriophages in *Staphylococcus aureus* host adaption. Microb. Physiol..

[103] Hayashida A., Bartlett A.H., Foster T.J., Park P.W. (2009). *Staphylococcus aureus* beta-toxin induces lung injury through syndecan-1. Am. J. Pathol..

[104] Katayama Y., Baba T., Sekine M., Fukuda M., Hiramatsu K. (2013). Beta-hemolysin promotes skin colonization by *Staphylococcus aureus*. J. Bacteriol..

[105] Rasigade J.P., Laurent F., Lina G., Meugnier H., Bes M., Vandenesch F., Etienne J., Tristan A. (2010). Global distribution and evolution of panton-valentine leukocidin-positive methicillin-susceptible *Staphylococcus aureus*, 1981–2007. J. Infect. Dis..

[106] Valle D.L., Paclibare P.A., Cabrera E.C., Rivera W.L. (2016). Molecular and phenotypic characterization of methicillin-resistant *Staphylococcus aureus* isolates from a tertiary hospital in the Philippines. Trop. Med. Health.

[107] International Working Group on the Classification of Staphylococcal Cassette Chromosome Elements (IWG-SCC) (2009). Classification of staphylococcal cassette chromosome mec (SCC*mec*): Guidelines for reporting novel SCCmec elements. Antimicrob. Agents Chemother..

[108] Uehara Y. (2022). Current status of Staphylococcal Cassette Chromosome *mec* (SCC*mec*). Antibiotics.

[109] Mairi A., Touati A., Pantel A., Yahiaoui Martinez A., Ahmim M., Sotto A., Dunyach-Remy C., Lavigne J.P. (2021). First Report of CC5-MRSA-IV-SCC*fus* “Maltese Clone” in Bat Guano. Microorganisms.

[110] Aouati H., Hadjadj L., Aouati F., Agabou A., Ben Khedher M., Bousseboua H., Bentchouala C., Rolain J.M., Diene S.M. (2021). Emergence of methicillin-resistant *Staphylococcus aureus* ST239/241 *SCCmec*-III mercury in Eastern Algeria. Pathogens.

[111] Fusco V., Quero G.M., Morea M., Blaiotta G., Visconti A. (2011). Rapid and reliable identification of *Staphylococcus aureus* harbouring the enterotoxin gene cluster (*egc*) and quantitative detection in raw milk by real time PCR. Int. J. Food Microbiol..

[112] Bolger A.M., Lohse M., Usadel B. (2014). Trimmomatic: A flexible trimmer for Illumina sequence data. Bioinformatics.

[113] Bankevich A., Nurk S., Antipov D., Gurevich A.A., Dvorkin M., Kulikov A.S., Lesin V.M., Nikolenko S.I., Pham S., Prjibelski A.D. (2021). SPAdes: A new genome assembly algorithm and its applications to single-cell sequencing. J. Comput. Biol..

[114] Rodriguez-R L.M., Gunturu S., Harvey W.T., Rosselló-Mora R., Tiedje J.M., Cole J.R., Konstantinidis K.T. (2018). The Microbial Genomes Atlas (MiGA) webserver: Taxonomic and gene diversity analysis of Archaea and Bacteria at the whole genome level. Nucleic Acids Res..

[115] Seemann T. (2014). Prokka: Rapid prokaryotic genome annotation. Bioinformatics.

[116] Tatusova T., DiCuccio M., Badretdin A., Chetvernin V., Nawrocki E.P., Zaslavsky L., Lomsadze A., Pruitt K.D., Borodovsky M., Ostell J. (2016). NCBI prokaryotic genome annotation pipeline. Nucleic Acids Res..

[117] BLAST. http://blast.ncbi.nlm.nih.gov/Blast.cgi.

[118] Page A.J., Cummins C.A., Hunt M., Wong V.K., Reuter S., Holden M.T., Fookes M., Falush D., Keane J.A., Parkhill J. (2015). Roary: Rapid large-scale prokaryote pan genome analysis. Bioinformatics.

[119] Hadfield J., Croucher N.J., Goater R.J., Abudahab K., Aanensen D.M., Harris S.R. (2018). Phandango: An interactive viewer for bacterial population genomics. Bioinformatics.

[120] Goris J., Konstantinidis K.T., Klappenbach J.A., Coenye T., Vandamme P., Tiedje J.M. (2007). DNA-DNA hybridization values and their relationship to whole-genome sequence similarities. Int. J. Syst. Evol. Microbiol..

[121] Rodriguez-R L.M., Konstantinidis K.T. (2016). The enveomics collection: A toolbox for specialized analyses of microbial genomes and metagenomes. Peer J..

[122] BV-BRC: Bacterial And Viral Bioinformatics Resource Center. https://www.bv-brc.org/.

[123] Stamatakis A. (2014). RAxML version 8: A tool for phylogenetic analysis and post-analysis of large phylogenies. Bioinformatics.

[124] Letunic I., Bork P. (2019). Interactive Tree of Life (iTOL) v4: Recent updates and new developments. Nucleic Acids Res..

[125] MLST. https://cge.cbs.dtu.dk/services/MLST.

[126] Larsen M.V., Cosentino S., Rasmussen S., Friis C., Hasman H., Marvig R.L., Jelsbak L., Sicheritz-Pontén T., Ussery D.W., Aarestrup F.M. (2012). Multilocus sequence typing of total genome sequenced bacteria. Clin. Micobiol..

[127] spaTyper. https://cge.cbs.dtu.dk/services/spaTyper.

[128] Bartels M.D., Petersen A., Worning P., Nielsen J.B., Larner-Svensson H., Johansen H.K., Andersen L.P., Jarløv J.O., Boye K., Larsen A.R. (2014). Comparing whole-genome sequencing with Sanger sequencing for spa typing of methicillin-resistant *Staphylococcus aureus*. J. Clin. Microbiol..

[129] Davis J.J., Boisvert S., Brettin T., Kenyon R.W., Mao C., Olson R., Overbeek R., Santerre J., Shukla M., Wattam A.R. (2016). Antimicrobial Resistance Prediction in PATRIC and RAST. Sci Rep..

[130] Liu B., Zheng D., Jin Q., Chen L., Yang J. (2019). VFDB 2019: A comparative pathogenomic platform with an interactive web interface. Nucleic Acids Res..

[131] VFDB. http://www.mgc.ac.cn/VFs/.

[132] Babicki S., Arndt D., Marcu A., Liang Y., Grant J.R., Maciejewski A., Wishart D.S. (2016). Heatmapper: Web-enabled heat mapping for all. Nucleic Acids Res..

[133] Merda D., Felten A., Vingadassalon N., Denayer S., Titouche Y., Decastelli L., Hickey B., Kourtis C., Daskalov H., Mistou M.Y. (2020). NAuRA: Genomic Tool to Identify Staphylococcal Enterotoxins in *Staphylococcus aureus* Strains Responsible for FoodBorne Outbreaks. Front. Microbiol..

[134] SCCmecFinder. https://cge.cbs.dtu.dk/services/SCCmecFinder-1.2.

[135] Kaya H., Hasman H., Larsen J., Stegger M., Johannesen T.B., Allesøe R.L., Lemvigh C.K., Aarestrup F.M., Lund O., Larsen A.R. (2018). SCCmecFinder, a web-based tool for typing of Staphylococcal Cassette Chromosome mec in *Staphylococcus aureus* using whole-genome sequence data. mSphere.

[136] Schubert J., Podkowik M., Bystroń J., Bania J. (2016). Production of staphylococcal enterotoxins in microbial broth and milk by *Staphylococcus aureus* strains harboring *seh* gene. Int. J. Food Microbiol..

[137] Schubert J., Podkowik M., Bystroń J., Bania J. (2017). Production of staphylococcal enterotoxins D and R in milk and meat juice by *Staphylococcus aureus* strains. Foodborne Pathog. Dis..

[138] Valihrach L., Alibayov B., Zdenkova K., Demnerova K. (2014). Expression and production of staphylococcal enterotoxin C is substantially reduced in milk. Food Microbiol..

[139] Pfaffl M.W. (2001). A new mathematical model for relative quantification in real-time RT-PCR. Nucleic Acids Res..

