# Supplementary material for: First Genome-Based Characterisation and Staphylococcal Enterotoxin Production Ability of Methicillin-Susceptible and Methicillin-Resistant Staphylococcus aureus Strains Isolated from Ready-to-Eat Foods in Algiers (Algeria)
Source: Toxins (Basel). 2022 Oct 25;14(11):731. doi: 10.3390/toxins14110731 (PMC9694651; doi:10.3390/toxins14110731)
Supplement: Supplementary file 1 [file toxins-14-00731-s001.zip › Supplementary Table S1_Genomic features.pdf]

Supplementary Table S1. Genomic features of the *S. aureus* strains sequenced in this study

Isolate	Genome size (bp)	GC (%)	N. of contigs	Completeness % (essential genes found)	Quality	Contig N50 (bp)	Genes (total)	Genes (coding)	Coding density	Genes (RNA)	rRNAs	tRNAs	ncRNAs	Pseudo Genes (total)
**SA01**	2,702,948	32.69	27	99.1%	80.1% (excellent)	211,213	2,657	2,557	83.99%	42	1, 1, 1 (5S, 16S, 23S)	35	4	58
**SA02**	2,794,901	32.73	29	95.5%	77.5% (high)	213,609	2,780	2,654	84.11%	42	2, 1, 1 (5S, 16S, 23S)	34	4	84
**SA04**	2,785,376	32.69	17	99.1%	80.1% (excellent)	730,703	2,765	2,648	83.86%	55	1, 4, 1 (5S, 16S, 23S)	45	4	62
**SA07**	2,666,473	32.67	31	99.1%	80.1% (excellent)	215,162	2,601	2,502	83.71%	43	2, 1, 1 (5S, 16S, 23S)	35	4	56
**SA08**	2,769,312	32.67	20	99.1%	80.1% (excellent)	308,007	2,768	2,645	83.86%	55	2, 1, 2 (5S, 16S, 23S)	46	4	68
**SA10**	2,762,907	32.67	34	99.1%	80.1% (excellent)	172,101	2,697	2,576	83.83%	55	1, 2, 1 (5S, 16S, 23S)	47	4	66
**SA18**	2,740,622	32.70	28	99.1%	80.1% (excellent)	286,872	2,721	2,607	84.06%	44	1, 2, 2 (5S, 16S, 23S)	35	4	70
**SA20**	2,759,779	32.56	33	99.1%	80.1% (excellent)	284,374	2,712	2,595	83.59%	34	2, 1, 1 (5S, 16S, 23S)	34	4	75
**SA24**	2,710,202	32.67	31	99.1%	80.1% (excellent)	212,794	2,667	2,575	83.97%	44	2, 1, 2 (5S, 16S, 23S)	35	4	48
**SA46**	2,811,261	32.78	43	99.1%	80.1% (excellent)	248,046	2,804	2,698	84.27%	44	1, 2, 1 (5S, 16S, 23S)	36	4	62
**SA51**	2,776,461	32.70	35	99.1%	80.1% (excellent)	209,034	2,765	2,659	84.06%	44	2, 1, 1 (5S, 16S, 23S)	36	4	62
**SA82**	2,726,122	32.70	40	99.1%	80.1% (excellent)	150,980	2,690	2,556	84.05%	55	2, 1, 3 (5S, 16S, 23S)	45	4	79
