# Supplementary material for: First Genome-Based Characterisation and Staphylococcal Enterotoxin Production Ability of Methicillin-Susceptible and Methicillin-Resistant Staphylococcus aureus Strains Isolated from Ready-to-Eat Foods in Algiers (Algeria)
Source: Toxins (Basel). 2022 Oct 25;14(11):731. doi: 10.3390/toxins14110731 (PMC9694651; doi:10.3390/toxins14110731)
Supplement: Supplementary file 1 [file toxins-14-00731-s001.zip › Supplementary Table S2_MLST allelic profile.pdf]

Supplementary Table S2. Allelic profile and MLST of the sequenced *S. aureus* strains

strain	*arcC*	*aro*E	*glp*F	*gm*K	*pta*	*tpi*	*yqil*	ST
SA01	13	13	1	1	12	11	13	15
SA02	1	4	1	4	12	1	10	5
SA04	1	1	1	1	1	1	1	1
SA07	13	1025[a]	1	1	12	11	13	7199[b]
SA08	4	1	4	1	5	5	4	25
SA10	3	1	14	15	11	19	3	101
SA18	3	1	1	1	1	5	3	97
SA20	3	3	1	1	4	4	3	8
SA24	1	4	1	8	4	4	954[a]	7200[b]
SA46	1	3	1	14	11	51	10	80
SA51	1	4	1	4	12	1	10	5
SA82	7	6	1	5	8	8	6	22

[a]Novel allele; [b]Novel ST.
