# Supplementary material for: First Genome-Based Characterisation and Staphylococcal Enterotoxin Production Ability of Methicillin-Susceptible and Methicillin-Resistant Staphylococcus aureus Strains Isolated from Ready-to-Eat Foods in Algiers (Algeria)
Source: Toxins (Basel). 2022 Oct 25;14(11):731. doi: 10.3390/toxins14110731 (PMC9694651; doi:10.3390/toxins14110731)
Supplement: Supplementary file 1 [file toxins-14-00731-s001.zip › Supplementary Table S3_spa_typing.pdf]

Supplementary Table S3. Spa typing of *S. aureus* strains

	spa Type	Repeats	Contig	Position	Orientation
SA01	t084	07-23-12-34-34-12-12-23-02-12-23	NODE_8_length_118714_cov_88.696747	52873-53174	minus
SA02	t002	26-23-17-34-17-20-17-12-17-16	NODE_1_length_533168_cov_112.199420	502749-503026	minus
SA04	t127	07-23-21-16-34-33-13	NODE_2_length_730703_cov_73.732725	346069-346274	minus
SA07	t094	07-23-12-34-34-12-12-23	NODE_8_length_146928_cov_75.988083	139442-139671	plus
SA08	t078	04-21-12-41-20-17-12-12-17	NODE_4_length_308007_cov_76.643097	144219-144472	plus
SA10	t4679	04-13-20-17-12-17-17	NODE_3_length_313697_cov_80.644675	247489-247694	minus
SA18	t9432	26-23-21-17-34-34-34-33-34	NODE_2_length_557737_cov_98.654831	144097-144350	plus
SA20	t2953	11-12-21-17-34-24-34-22-25-25	NODE_10_length_94764_cov_98.480647	41304-41581	plus
SA24	t3092	07-23-12-21-12-17-20-17-12-17	NODE_1_length_446589_cov_97.377358	306504-306781	minus
SA46	t044	07-23-12-34-34-33-34	NODE_7_length_183493_cov_99.007458	139502-139707	plus
SA51	t002	26-23-17-34-17-20-17-12-17-16	NODE_1_length_511254_cov_83.330293	367765-368042	minus
SA82	t223	26-23-13-23-05-17-25-17-25-16-28	NODE_17_length_45595_cov_46.844875	15640-15941	minus
