# Supplementary material for: First Genome-Based Characterisation and Staphylococcal Enterotoxin Production Ability of Methicillin-Susceptible and Methicillin-Resistant Staphylococcus aureus Strains Isolated from Ready-to-Eat Foods in Algiers (Algeria)
Source: Toxins (Basel). 2022 Oct 25;14(11):731. doi: 10.3390/toxins14110731 (PMC9694651; doi:10.3390/toxins14110731)
Supplement: Supplementary file 1 [file toxins-14-00731-s001.zip › Supplementary Table S6_Roary statistics.pdf]

Supplementary Table S6. Roary statistics

Core genes	(99% <= strains <= 100%)	1596
Soft core genes	(95% <= strains < 99%)	261
Shell genes	(15% <= strains < 95%)	1323
Cloud genes	(0% <= strains < 15%)	4357
Total genes	(0% <= strains <= 100%)	7537
