# Supplementary figures and images for: First Genome-Based Characterisation and Staphylococcal Enterotoxin Production Ability of Methicillin-Susceptible and Methicillin-Resistant Staphylococcus aureus Strains Isolated from Ready-to-Eat Foods in Algiers (Algeria)
Source: Toxins (Basel). 2022 Oct 25;14(11):731. doi: 10.3390/toxins14110731 (PMC9694651; doi:10.3390/toxins14110731)

Tree scale: 0.1

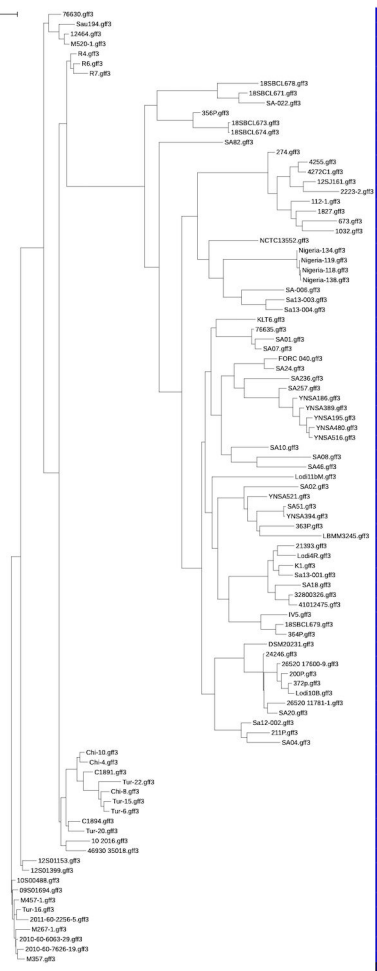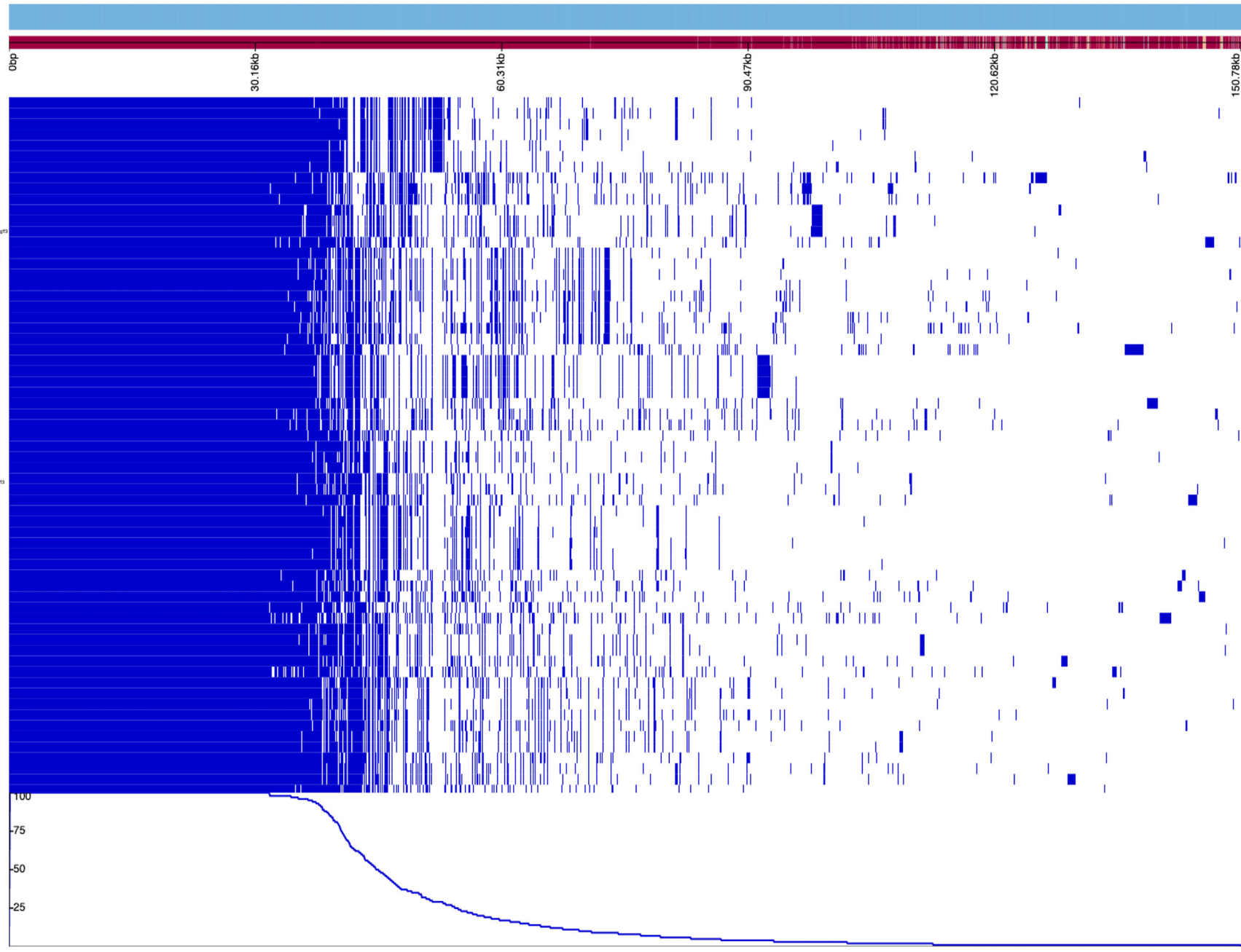

Supplement: Supplementary file 1 [file toxins-14-00731-s001.zip › Supplementary Fig. S1_Pan-genome.pdf]

**SA02**

vSaß type I

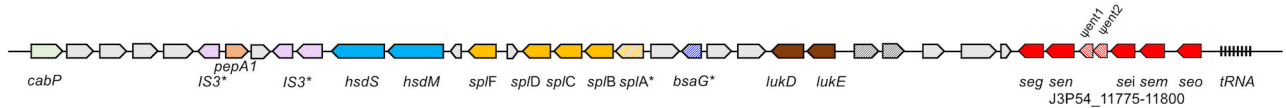

**SA51**

vSaß type I

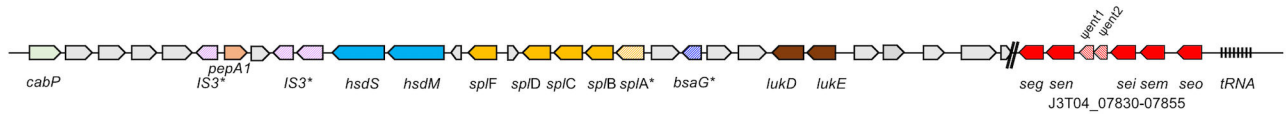

**SA24**

vSaß type I

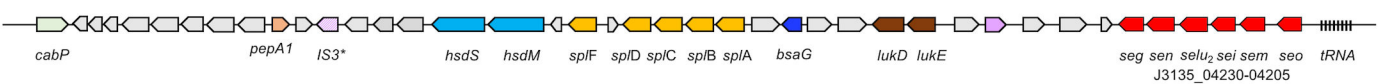

**SA08**

vSaß type IV

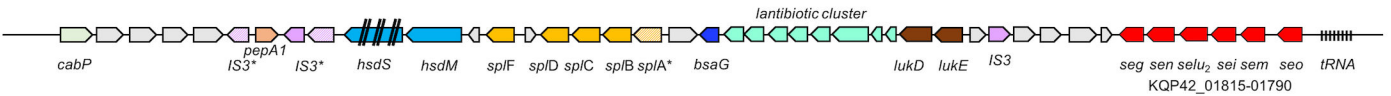

**SA82**

vSaß type III

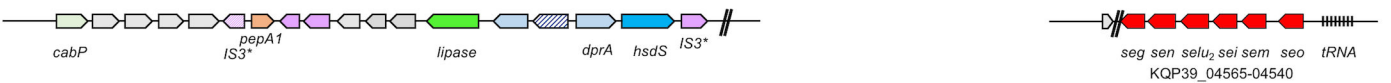

Supplement: Supplementary file 1 [file toxins-14-00731-s001.zip › Supplementary Fig.2_egc.pdf]
